# *Operando* pair distribution function analysis of nanocrystalline functional materials: the case of TiO_2_-bronze nanocrystals in Li-ion battery electrodes

**DOI:** 10.1107/S1600576724005624

**Published:** 2024-07-29

**Authors:** Martin A. Karlsen, Jonas Billet, Songsheng Tao, Isabel Van Driessche, Simon J. L. Billinge, Dorthe B. Ravnsbæk

**Affiliations:** a Department of Chemistry, Aarhus University, DK-8000Aarhus C, Denmark; b Department of Chemistry, Ghent University, 9000Ghent, Belgium; c Department of Applied Physics and Applied Mathematics with Materials Science and Engineering, Columbia University, New York, NY10027, USA; Australian Synchrotron, ANSTO, Australia

**Keywords:** *operando*, pair distribution function analysis, nanocrystalline functional materials, TiO_2_-bronze, Li-ion batteries

## Abstract

Structural modelling of *operando* pair distribution function (PDF) data of complex functional materials can be highly challenging. To aid the understanding of complex *operando* PDF data, this article demonstrates a toolbox for PDF analysis. The tools include denoising using principal component analysis together with the *structureMining*, *similarityMapping* and *nmfMapping* apps available through the online service ‘PDF in the cloud’ (*PDFitc*, https://pdfitc.org/). The toolbox is used for both *ex situ* and *operando* PDF data for 3 nm TiO_2_-bronze nanocrystals, which function as the active electrode material in a Li-ion battery. The tools enable structural modelling of the *ex situ* and *operando* PDF data, revealing two pristine TiO_2_ phases (bronze and anatase) and two lithiated Li_*x*_TiO_2_ phases (lithiated versions of bronze and anatase), and the phase evolution during galvanostatic cycling is characterized.

## Abstract 2

This article provides a demonstration of tools for *operando* pair distribution function analysis of nanocrystalline functional materials. The particular case used is 3 nm TiO_2_-bronze nanocrystals as active electrode material in a Li-ion battery.

## 1. Introduction

For many electrode materials for rechargeable batteries, crystallinity, *i.e.* long-range structural order, has been thought of as a prerequisite (Whittingham, 2004 ▸; Goodenough & Kim, 2010 ▸; Christensen & Ravnsbæk, 2021 ▸). However, in recent years, it has been realized that crystalline defects, nanosizing, amorphization *etc*. may be beneficial for electrochemical performance (Uchaker *et al.*, 2014 ▸; Sheng *et al.*, 2014 ▸; Chae *et al.*, 2014 ▸; Hua *et al.*, 2017 ▸; Luo *et al.*, 2017 ▸; Wang *et al.*, 2018 ▸; Christensen *et al.*, 2019*a* ▸; Christensen & Ravnsbæk, 2021 ▸). Insights into structural transformations of battery electrodes are obtainable through *operando* experiments (Chianelli *et al.*, 1978 ▸, 1979 ▸; Latroche *et al.*, 1992 ▸). For crystalline phases, *operando* powder X-ray diffraction (PXRD) and Rietveld analysis (Rietveld, 1969 ▸) are also applicable to electrode materials for batteries (Tarascon *et al.*, 1999 ▸; Bak *et al.*, 2018 ▸). If the length of structural coherence of a phase shortens, PXRD and Rietveld analysis are no longer ideal tools for extracting information on the atomic structure. Instead, this information can be extracted through X-ray total scattering (XTS) and atomic pair distribution function (PDF) analysis (Billinge & Kanatzidis, 2004 ▸; Billinge & Levin, 2007 ▸; Billinge, 2009 ▸; Egami & Billinge, 2012 ▸). Using PDF analysis, phase transitions involving non-crystalline phases under dynamic conditions may be explored through *operando* XTS combined with PDF analysis (Hua *et al.*, 2017 ▸; Christensen *et al.*, 2018 ▸, 2019*a* ▸,*b* ▸). As battery electrodes are multicomponent systems containing active material, conductive carbon and polymeric binder, PDF data for battery electrodes are usually highly complex and therefore highly challenging to model. To assist the structural modelling, we demonstrate multiple types of model-free analyses to gain insights into *operando* PDF data for nanocrystalline battery electrodes.

This study is concerned with TiO_2_-based electrode materials for rechargeable Li-ion batteries. The family of titanium dioxide, TiO_2_, polymorphs is large and diverse. The family members share the common building block of TiO_6_ octahedra, which are connected in different ways, giving rise to the various polymorphs (Liu *et al.*, 2013 ▸; Aravindan *et al.*, 2015 ▸). Examples of TiO_2_ polymorphs include anatase (Cromer & Herrington, 1955 ▸), rutile (Cromer & Herrington, 1955 ▸), brookite (Pauling & Sturdivant, 1928 ▸), bronze (Marchand *et al.*, 1980 ▸), columbite (Simons & Dachille, 1967 ▸), hollandite (Latroche *et al.*, 1989 ▸), ramsdellite (Akimoto *et al.*, 1994 ▸), baddeleyite (Sato *et al.*, 1991 ▸), TiO_2_-O-I (Dubrovinskaia *et al.*, 2001 ▸) and TiO_2_-O-II (Dubrovinsky *et al.*, 2001 ▸), where the first four polymorphs are common at ambient temperatures and pressures. The diversity of the TiO_2_ poymorphs results in versatile use as functional materials, such as wide-band-gap semiconductors (∼3.2 eV) (Elmouwahidi *et al.*, 2018 ▸) with spectral activity in the ultraviolet (UV) domain (Gonçalves *et al.*, 2008 ▸). TiO_2_ materials also find use as photovoltaics, *e.g.* dye sensitized photovoltaic modules (Kay & Grätzel, 1996 ▸) and solar cells (Gonçalves *et al.*, 2008 ▸), photocatalysts (Fujishima *et al.*, 2008 ▸; Fresno *et al.*, 2014 ▸), supercapacitors (Elmouwahidi *et al.*, 2018 ▸), and electrochemical storage. Regarding electrochemical storage, TiO_2_ materials have been widely explored as intercalation-type electrode materials for Li-ion batteries. The theoretical gravimetric capacity of TiO_2_ materials in Li-ion batteries reaches 335 mAh g^−1^ for intercalation of one equivalent of Li^+^, making TiO_2_ materials promising alternatives to the commercial anode material Li_4_Ti_5_O_12_ (175 mAh g^−1^) and carbon-based anodes (372 mAh g^−1^). In addition to a high theoretical gravimetric capacity, TiO_2_ materials are also attractive materials due to the low production cost and low environmental impact (Deng *et al.*, 2009 ▸; Yang *et al.*, 2009 ▸; Fröschl *et al.*, 2012 ▸; Christensen *et al.*, 2019*a* ▸).

**Figure 1:**
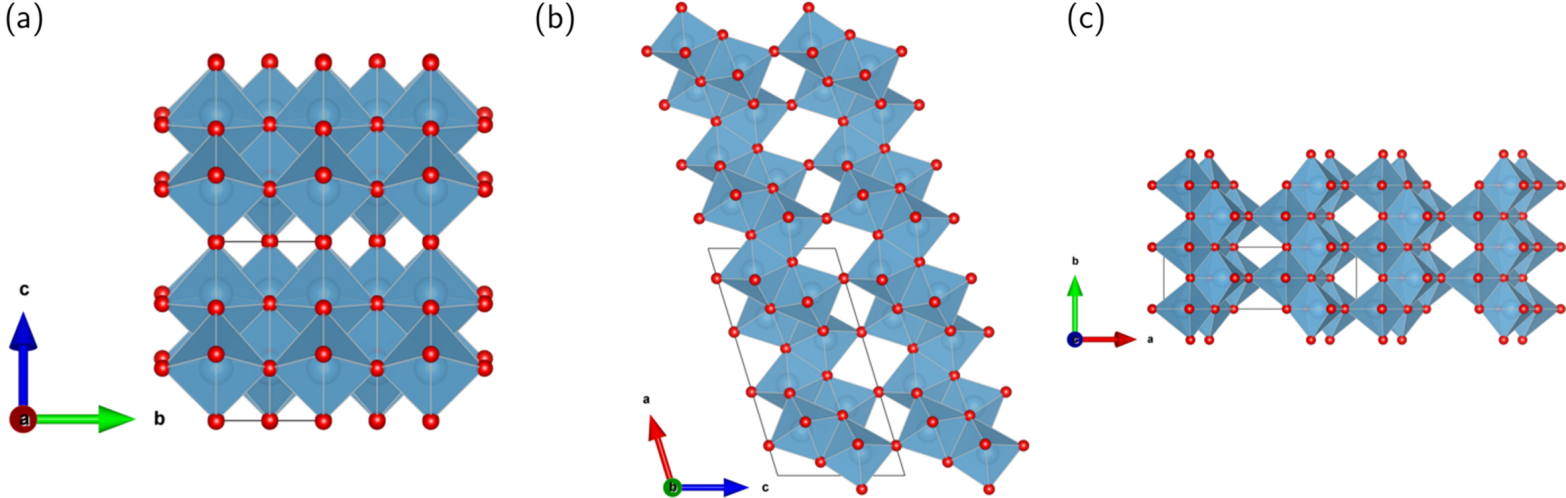
Visualization of the TiO_2_-bronze structure. The 2 × 2 × 2 monoclinic (*C*2/*m*) unit cells are displayed along the crystallographic (*a*) *a*, (*b*) *b* and (*c*) *c* axes. Ti and O atoms are shown in light blue and red, respectively.

Among the TiO_2_ polymorphs, the bronze polymorph has received additional attention due its high operation power and capacity performances (Gao *et al.*, 2019 ▸). Compared with commercial graphite anodes, TiO_2_-bronze also offers higher operation safety through its higher discharge voltage plateau (>1.7 V versus Li/Li^+^) (Liang *et al.*, 2022 ▸). Its monoclinic unit cell, depicted along the three crystallographic axes in Fig. 1 ▸, belongs to the *C*2/*m* space group. The network of edge- and corner-sharing TiO_6_ octahedra has channels along the *b* axis, *i.e.* the [010] direction, suitable for ion intercalation (Arrouvel *et al.*, 2009 ▸; Pham *et al.*, 2021 ▸).

### 1.1. Recent tools for PDF analysis

To assist scientists in their PDF analyses, the service ‘PDF in the cloud’ (*PDFitc*) is available at https://pdfitc.org/ (Yang *et al.*, 2021 ▸), which offers a number of different apps for PDF analysis. Herein, we demonstrate the use of the *structureMining*, *similarityMapping* and *nmfMapping* apps available at *PDFitc*. We also make use of principal component analysis (PCA, not available through *PDFitc*) for denoising of the *operando* data. The various tools greatly assist our analysis of both *ex situ* and *operando* PDF data and will be described briefly below.

#### 1.1.1. structureMining

The *structureMining* app (Yang *et al.*, 2020 ▸) offers phase identification for PDFs. Phase identification or ‘fingerprinting’ is common for PXRD data in many laboratories, since the establishment of the Hanawalt file (Hanawalt *et al.*, 1938 ▸) in the first part of the 20th century. This has been followed by a number of descendants, including the Powder Diffraction File (Gates-Rector & Blanton, 2019 ▸).

To run a search query, the user uploads an experimental (or simulated) PDF together with relevant metadata like compositional and experimental details. On completing the search query, the *structureMining* app returns a list of crystallographic information files (.cif) (Hall *et al.*, 1991 ▸). The rank of each .cif file is based on the weighted residual value, *R*_w_, when refining the user-uploaded PDF data using a structural model based on the .cif file. This allows the user to base a quantitative analysis, *i.e.* PDF refinement, on one or more of the .cif files returned by the app.

The *structureMining* app complements ‘traditional’ phase identification from PXRD data just as PDF complements PXRD. Whenever the length of structural coherence of a phase in a material is so short that no sharp Bragg peaks can be used for phase identification in reciprocal space, PDF data and the *structureMining* app still allow for phase identification. In the present case, the primary phase of the TiO_2_ nanocrystals can be identified in reciprocal space but the secondary phase with even shorter length of structural coherence is exclusively identified in real space from the PDF data by the *structureMining* app.

#### 1.1.2. Principal component analysis

Inspired by the *nmfMapping* app (Section 1.1.4), we make use of PCA to denoise the experimental *operando* PDF data. The denoising cleans the experimental signal, improving visualization and thereby qualitative inspection of the *operando* PDF data. The data otherwise suffer from decreasing signal-to-noise ratio with *r*, due to damping of the PDF. For the present case of nanocrystals, the PDF is heavily damped because of both the instrument and the nanosized samples such that the signal-to-noise ratio becomes a challenge even at relatively low *r* values. In the present case, the various phases encountered possess highly similar local structure owing to the common building blocks of TiO_6_ octahedra. This makes it highly challenging to distinguish the various phases at low *r* and one must move further out in *r* for the denoising to pay off.

PCA is preferred for denoising over non-negative matrix factorization (NMF), where the PDF data need to be shifted on the *G* scale both before and after NMF analysis. In contrast, PCA is directly applicable to the PDF data with minimal user interference.

The denoised data are solely used qualitatively for visual inspection, where the denoising cleans the signal and improves the visualization significantly. The denoised data are not used for the structural modelling. A possible pay-off if using denoised data for the structural modelling would be decreased residuals with no change in the structural parameters. On the other hand, there will always be a risk of over-filtering of the experimental noise that could affect the structural refinement. Due to this risk assessment, the denoised data are not used for the structural modelling.

#### 1.1.3. similarityMapping

The *similarityMapping* app is meant for probing similarity for a series of PDFs uploaded by the user. During a query, the app runs a Pearson correlation analysis (Pearson & Galton, 1895 ▸) for the set of PDFs uploaded by the user. The Pearson correlation coefficient of two PDFs becomes a measure of similarity between the PDFs. Then, the similarity of two PDFs is interpreted as a measure of the similarity between the phase contents of the materials from which the PDFs originate. The analysis is purely statistical in nature and therefore model free, quick and straightforward. For *operando* data, the *similarityMapping* can be used to identify the onset of phase transitions but also whether phase evolution appears to possess solid-solution or two-phase characteristics.

#### 1.1.4. nmfMapping

The *nmfMapping* app (Liu *et al.*, 2021 ▸; Thatcher *et al.*, 2022 ▸) offers NMF analysis for a series of PDFs. The app decomposes a series of PDFs and describes the trends in the data with as few components as possible. Being an unsupervised machine learning technique, NMF analysis shares some characteristics with PCA. The differences between NMF and PCA include the nature of the constraints of the matrix decomposition. The non-negative constraint of the matrix decomposition for the NMF analysis means that the (normalized) NMF weights can be interpreted as fractions of the total scattering signal, which is directly related to the phase fractions of the material. Therefore, the number of components from NMF analysis can provide a hint as to the number of phases present during an *operando* experiment and the behaviour of the NMF weights offers insights into the evolution of the phases that the NMF components present. As NMF is purely statistical in nature, one can easily obtain outputs without any physical significance. However, when used with care, NMF analysis can provide physically meaningful outputs. The number of NMF components can be interpreted as representing the number of phases present and the behaviour of the NMF weights can be interpreted as representing the behaviour of phase fractions. The NMF weights then represent the fractions of the total scattering signal and not directly molar or mass fractions.

## 2. Methods

### 2.1. Nanocrystal synthesis

Two batches of TiO_2_ nanocrystals were studied. The first batch was used for *ex situ* characterization, including chemical lithiation (Section 2.2). The second batch was used for *ex situ* and *operando* characterization. The TiO_2_ nanocrystals were synthesized using a microwave setup as previously described by Billet *et al.* (2018 ▸). The molar concentration of Ti was 0.244 *M*, the molar concentration of glycolic acid was 0.25 *M* and that of sulfuric acid was 0.72 *M*. The reaction mixture was treated at 130°C for 5 min. Finally, the nanocrystals were washed three times with water.

### 2.2. Chemical lithiation

For the first batch of TiO_2_ nanocrystals, the material was dried overnight under vacuum at 60°C. Nanocrystals (50 mg) were suspended in anhydrous heptane. To ensure complete lithiation, three equivalents (7 ml) of *N*-butyllithium (2.7 *M* in heptane, Sigma–Aldrich) were added dropwise to the sus­pen­sion under magnetic stirring in an Ar-filled atmosphere. The mixture was left to react for 2 days. The remaining liquid was removed and the powder was washed in heptane three times and dried. To obtain a fine powder, the chemically lithiated materials were mortared using an agate mortar and pestle.

### 2.3. Electrode fabrication

For 200 mg of cathode composite, 60 wt% active material (120 mg TiO_2_ nanocrystals, batch two), 30 wt% conductive carbon [30 mg SuperP C45 (Imerys) and 30 mg acetylene black (VXC72, Cabot Corp.)] and 10 wt% polymeric binder [20 mg polyvinylidene fluoride (PVDF, Kynar, Arkena)] were used. The active material and the conductive carbon were weighed separately, whereas the polymeric binder was obtained from a 4 wt% *N*-methyl-2-pyrrolidone (NMP, 99.5%, anhydrous, Sigma–Aldrich) solution. The active material, conductive carbon and PVDF/NMP solution were mixed in a plastic vial with a Teflon ball using a vortex mixer to obtain a slurry. The slurry was poured onto a sheet of aluminium foil and spread on the foil using the ‘doctor blade’ method. The coated aluminium foil was left to dry in the fumehood overnight at 60°C. The drying ended with 1 h at 90°C to ensure complete evaporation of the NMP. The dry cathode composite was scraped off the aluminium foil using a plastic spatula and mortared using an agate mortar and pestle to obtain a fine powder. The composite (8–12 mg) was uniaxially pressed into 7 mm Ø pellets at 1.8 ton for 1 min.

### 2.4. Electrochemical cell assembly

For the *operando*X-ray total scattering studies, the AMPIX electrochemical cell (Borkiewicz *et al.*, 2012 ▸) was used. The half-cell was assembled in an Ar-filled glovebox with a 11.259 mg cathode pellet (6.755 mg TiO_2_-bronze nanocrystals) bottommost. A 12 mm Ø Whatman GF/B separator was put on top of the cathode pellet. The separator was wetted with seven drops of 1 *M* LiPF_6_ in ethylene carbonate: dimethyl carbonate, 1:1 *v*/*v* (99.9%, Solvionic) using a 1 mL Pasteur pipette. A metallic Li anode was placed topmost. The Li anode was obtained by rolling lithium foil using a stainless steel rod. From the thinly rolled lithium foil, a 10 mm Ø disc was punched out.

### 2.5. Galvanostatic cycling

During the *operando*X-ray total scattering experiment, the electrochemical cell was galvanostatically cycled using a current density of 0.151 mA, corresponding to a C-rate of C/15.

### 2.6. Measurements

Pristine and chemically lithiated powders were characterized through *ex situ* PXRD and XTS. The electrode with 3 nm TiO_2_-bronze nanocrystrals as the active material was characterized through *operando* XTS. The synchrotron X-ray scattering experiments were conducted at beamline P02.1, PETRA III, DESY (Dippel *et al.*, 2015 ▸), using a PerkinElmer XRD1621 area detector. Experiments were conducted for two batches of TiO_2_-bronze nanocrystals. For the first batch, *ex situ* experiments for pristine and chemically lithiated material were conducted using an X-ray wavelength of 0.20721 Å. For the second batch, *ex situ* experiments were conducted for pristine material and for the cathode composite containing the active material, polymeric binder and conductive carbon (Section 2.3). For the second batch, an *operando* experiment was conducted using an X-ray wavelength of 0.20739 Å. The *ex situ* PXRD and XTS experiments were conducted using Kapton polyimide capillaries (1.0 mm inner diameter, Cole-Parmer). An empty capillary was used for the background measurement and highly crystalline CeO_2_ was used for calibration. For the *operando* XTS experiment, the AMPIX electrochemical cell was used. For the background measurement, an AMPIX cell containing separator wetted with electrolyte was used and an AMPIX electrochemical cell with highly crystalline CeO_2_ was used for calibration, including experimental geometry and instrumental contributions.

### 2.7. Data processing

For the *ex situ* experiments, the scattering data were processed using the *DAWN* software package (Filik *et al.*, 2017 ▸). The beamstop arm, dead pixels and over-exposed pixels were masked using the ‘fast masking’ tool. The ‘mask by coordinate’ feature, where lower and upper *Q* limits for the mask are stated by the user, was used to mask the beamstop at low *Q* and to mask incomplete Debye–Scherrer rings at high *Q*, effectively setting the range of azimuthal integration. For the *operando* data, the *Python Fast Azimuthal Integration* (*pyFAI*) software (Ashiotis *et al.*, 2015 ▸) was used. The calibration was done for a highly crystalline CeO_2_ standard. A detector mask was created by masking beamstop, beamstop arm, dead pixels and over-exposed pixels. To mask out single-crystal spots originating from the Li anode, an additional mask was created for each of the TiO_2_-bronze *operando* frames, using a Python-based automasking routine. To account for X-ray intensity fluctuations due to fluctuating current in the synchrotron storage ring during the *operando* experiment, the *operando* data were scaled in reciprocal space. For the *ex situ* and *operando* XTS data, background subtraction, normalization to obtain the total scattering structure function, *S*(*Q*), further reduction to obtain the reduced total scattering structure function, *F*(*Q*), and inverse Fourier transformation to obtain the reduced atomic pair distribution function, *G*(*r*), were done using the *PDFgetX3* algorithm (Juhás *et al.*, 2013 ▸) through the *xPDFsuite* (Yang *et al.*, 2015 ▸) program.

### 2.8. *Ex situ* PXRD and Rietveld analysis

For Rietveld analysis of the PXRD data for the pristine and chemically lithiated materials of batch one, the *TOPAS Academic V6* software (Coelho, 2018 ▸) was used. Crystallite sizes were estimated via the Scherrer method (Scherrer, 1918 ▸), using the volume-weighted column height (Dinnebier *et al.*, 2019 ▸). The instrumental contribution to the peak broadening was determined by refining the powder profile of highly crystalline CeO_2_. For the structural modelling of the pristine material, a monoclinic TiO_2_-bronze structure [space group *C*2/*m*, VO_2_(B) structure type (Théobald *et al.*, 1976 ▸), ICSD (Belsky *et al.*, 2002 ▸) collection code 41056 (Feist & Davies, 1992 ▸)] was used. For the structural modelling of the chemically lithiated material through Rietveld analysis, a single phase of lithiated TiO_2_-bronze [space group *C*2/*m*, ICSD 180011 (Armstrong *et al.*, 2010 ▸)] with composition Li_0.5_TiO_2_ was used. The results of the Rietveld analyses are presented in Section 3.1 and in Appendix *A* (in the supporting information).

### 2.9. *Ex situ* PDF modelling

**Figure 2:**
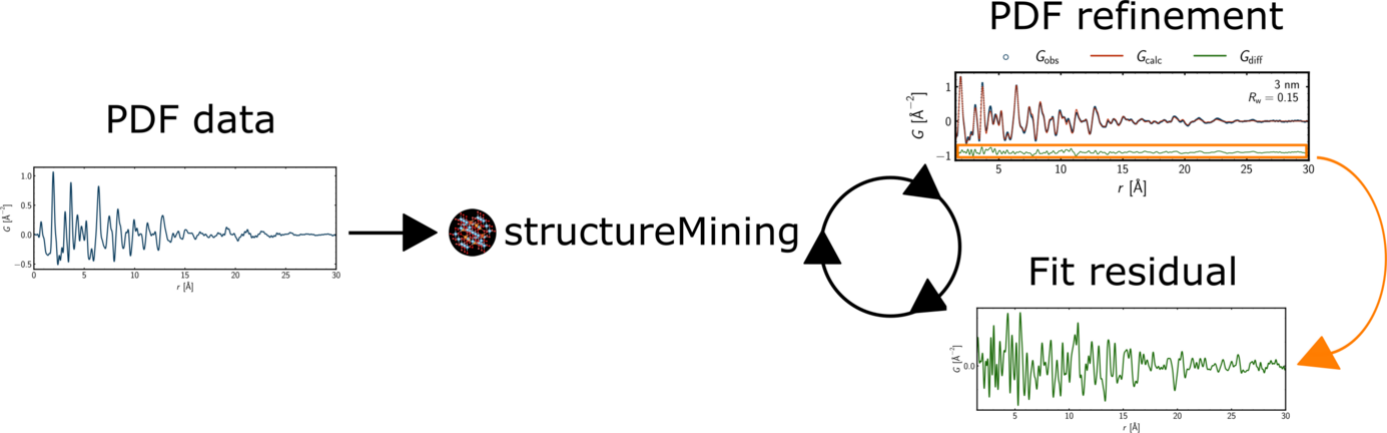
Workflow when using the *structureMining* app at *PDFitc*. PDF data are uploaded to the *structureMining* app. From the output of the app, a structural model is built and refined against the experimental data. If the model does not seem to describe the data sufficiently, the fit residual may be extracted and uploaded to the *structureMining* app, as if it were a PDF itself, *i.e.* with the same metadata as the original PDF. From the output of the *structureMining* app, a two-phase model is built and refined against the experimental data.

For analysis of the *ex situ* PDF data, the *diffpy-cmi* software (Juhás *et al.*, 2015 ▸) was used. Data for highly crystalline CeO_2_ were refined to obtain instrumental damping and broadening parameters, *Q*_damp_ and *Q*_broad_, which were then included in the analysis of the TiO_2_-bronze data as fixed parameters. For the refinements of the data for the TiO_2_-bronze nanocrystals, scale factors, unit-cell parameters, isotropic atomic displacement parameters (ADPs), *u*_iso_, and quadratic correlated motion parameters, δ_2_, were refined. As Li has a low atomic number and therefore a low X-ray scattering power, its contribution to the total scattering signal was expected to be low. In addition, possible disorder of Li would also broaden the scattering signal originating from Li. Therefore, the isotropic ADP of Li was not included in the refinement as a variable but included as a parameter fixed to a value of *u*_Li_ = 0.05 Å^2^. The coherent domain size for a spherical model was also refined to take nanosize into account. The atomic positions were refined as well, using space-group symmetry constraints. To stabilize the refinement, the atomic positions were refined using restraints of ±0.05 units of the relevant unit-cell length. Single-phase refinements were based on the output from the *structureMining* app at *PDFitc*. When single-phase refinements were insufficient for the PDF analyses, the PDF fit residual from *diffpy-cmi* was extracted and saved to a .gr file as if it were an experimental PDF. The extracted fit residual was then uploaded to the *structureMining* app with the same metadata as the original PDF. Using the *structureMining* output once again, two-phase refinements were conducted using *diffpy-cmi*. This approach is illustrated in Fig. 2 ▸ and will be described in detail in Section 3.2.

### 2.10. *Operando* PDF modelling

The *structureMining* app was not used *directly* for the *operando* data but only *indirectly* through the *ex situ* data, as the structures obtained from the modelling of the *ex situ* data were used as starting points for the modelling of the *operando* data.

To take possible non-subtracted signal from the glassy carbon windows of the AMPIX cell into account, together with the signal originating from the additive of the electrode composite, a modified graphite phase (not obtained through the *structureMining* app) was included in the analysis of the *operando* PDF data. To take stacking faults and turbostratic disorder into account, an anomalously high value of unity for the atomic displacement parameter along the *c* axis was used, *u*_33_ = 1.0. Graphically speaking, the interlayer atomic pair correlations are broadened so much that only the in-layer atomic pair correlations remain in the model. As the interlayer correlations are excluded for the modified graphite phase, the *c* lattice parameter is not included as a variable in the refinement; only the in-layer lattice parameter *a* is included. Instead of modelling the carbon signal, one could also subtract a scaled carbon signal from a refinement of the first scan of the pristine material. However, the modelling approach allows for small adjustments for the scale and in-plane lattice parameter such that the carbon contribution to the fit residual is minimized throughout the sequential refinement (Christensen *et al.*, 2019*a* ▸,*b* ▸).

### 2.11. Extracting time dependence of chemical components

To extract the time dependence of chemical components, *i.e.* the phase evolution, during the *operando* experiment, multiple model-free analyses were conducted.

#### 2.11.1. Principal component analysis

The sklearn.decomposition.PCA (Pedregosa *et al.*, 2011 ▸) Python (Van Rossum & Drake, 2009 ▸) module was used for the PCA. The primary purpose was to denoise the experimental *operando* data to improve qualitative visual inspection of the data. A part of the denoising process is to set the level of denoising. This was done by inspection of the (cumulated) explained variance ratio as a function of the number of components (Figs. D1 and D5, Appendix *D*, supporting information). Thereby, an indication of the number of components needed to describe the trends in the *operando* data was also obtained. This number was compared with that obtained through NMF analysis, as described in Section 2.11.3.

#### 2.11.2. *similarityMapping* (Pearson correlation analysis)

The *similarityMapping* app at *PDFitc* was used to inspect the similarity of the *operando* PDFs. The Pearson correlation coefficient (PCC) is the measure of similarity. Similar PDFs are expected to represent similar phase content and *vice versa* for dissimilar PDFs. Therefore, *similarityMapping* allows one to inspect phase evolution during the *operando* experiment, especially when making use of the complementary electrochemical information available from galvanostatic cycling. The onset of phase transitions will be evident as sudden dissimilarity between neighbouring PDFs. The nature of phase transitions, *e.g.* two-phase or solid-solution, will be evident as discrete or continuous changes of similarity, respectively. Disordering, *i.e.* shortening of the length of structural coherence, can be probed by conducting the correlation analysis for different *r* ranges (Figs. E1–E3, Appendix *E*, supporting information).

#### 2.11.3. *nmfMapping* (non-negative matrix factorization)

The *nmfMapping* app was used to identify the number of components needed to describe the trends in the *operando* data. This was done through the reconstruction error as a function of the number of components. Due to the non-negative constraint on the matrix decomposition, the behaviour of the NMF weights is likely to be physically meaningful. The NMF weights as a function of time during the galvanostatic cycling of the *operando* experiment provided information on the phase evolution. The behaviour of the NMF weights was used as guidance for when to include certain phases in the refinement of the *operando* data. In Appendix *F* (supporting information), it is shown how the NMF analysis can be done for both reciprocal-space *F*(*Q*) and real-space *G*(*r*) data by varying the number of components used for the matrix decomposition.

In Section 2.11.1, it is described how PCA was used for denoising the *operando* data, for which NMF could also be used. However, the PDFs must be shifted to a positive domain for NMF to be applicable. On the other hand, PCA was directly applicable. For the case of intensity versus momentum transfer, *I*(*Q*), the positive domain of the data means that NMF is directly applicable for use in denoising. Currently, the *nmfMapping* app only returns normalized weights, as these can be interpreted as fractions of the total scattering signal. Therefore, the reconstructed signal from the output of the *nmfMapping* app would not be on the same scale as the experimental data. This hampers direct comparison and validation of the reconstruction. However, one could use, *e.g.*, Pearson correlation analysis, which is insensitive to the scale of the data, just as it would be insensitive to the shifts introduced for making the domain of the PDFs positive for NMF to be applicable. If one had access to the non-normalized NMF weights, the NMF reconstruction could be on the same scale as the experimental *operando* data. If one also has access to the shift introduced to make the PDFs positive, direct comparison, *e.g.* through a difference plot, would be possible.

#### 2.11.4. Combining model-free analyses

**Figure 3:**
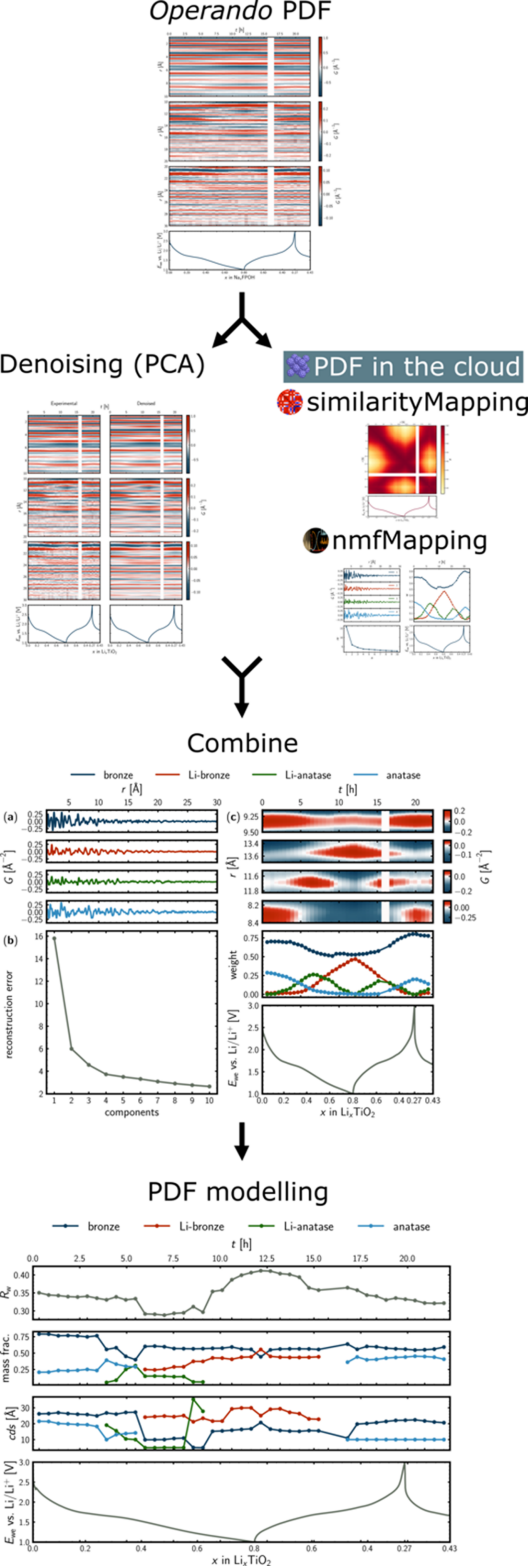
Workflow for use of various model-free analyses prior to structural modelling. The experimental *operando* PDF data are denoised using PCA. *PDFitc* offers model-free analyses through the *similarityMapping* and *nmfMapping* apps. The information obtained from the various model-free analyses is combined and guides the structural modelling.

Using the denoised data from the PCA, phase-specific atomic pair correlations were identified and their evolution lined up with the behaviour of the NMF weights. This underlines the physical significance of the NMF output. The workflow for extracting the time dependence of chemical components to use for modelling of the *operando* PDF data is illustrated in Fig. 3 ▸.

## 3. Results

### 3.1. *Ex situ* Rietveld analysis

The nanosized crystalline domains in the materials were readily evident from the very broad reflections in the diffraction patterns. Fits from Rietveld analyses of the *ex situ* PXRD data of pristine and chemically lithiated materials of batch one can be found as Fig. A1, Appendix *A*. The broadening of the peaks limits the amount of structural information extractable through Rietveld analysis, as uncertainties on the refined values are high. This underlines the need for PDF analysis. Further discussion of the Rietveld analysis results is provided in Appendix *A*.

### 3.2. *Ex situ* PDF analysis: *structureMining* and PDF modelling

The *structureMining* app at *PDFitc* was used to identify primary and secondary phases for *ex situ* PDF data of the pristine and chemically lithiated materials. For the pristine materials, the chemical composition was set to TiO_2_. For the chemically lithiated material, the queries were run for all lithium titanium oxides, Li_*x*_Ti_*y*_O_*z*_, by putting Li-Ti-O for the composition in the app. Initially, primary phases of the PDFs were identified. To identify secondary phases, the initial *structureMining* output was used as input model for the *diffpy-cmi* software and the model was refined. The *diffpy-cmi* fit residual was extracted and saved with the same metadata as the original PDF and then uploaded to the *structureMining* app, using the same composition as the initial search query. The second *structureMining* output was then incorporated into a two-phase model that was refined using *diffpy-cmi*. The workflow is illustrated in Fig. 2 ▸. The top five results of each *structureMining* search query are presented in Appendix *B* (supporting information), where the calculated PDFs are also presented.

**Figure 4:**
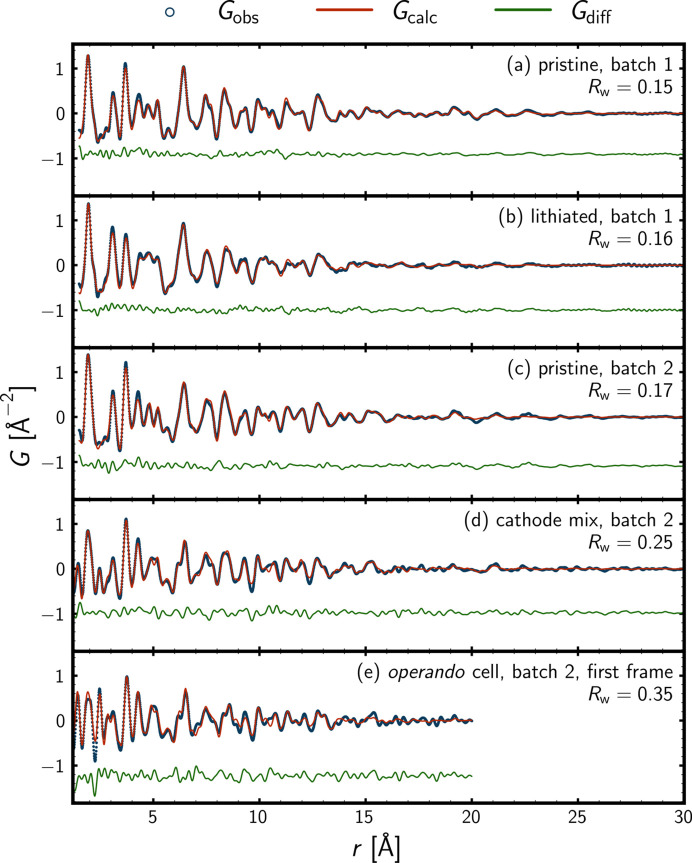
*Ex situ* PDF refinements. The observed PDF, *G*_obs_, is shown as blue circles, the calculated PDF, *G*_calc_, is shown as a red line, and the difference between the observed and calculated PDFs, *G*_diff_, is shown as a green line. The reduced atomic PDF, *G*, in Å^−2^ is shown as a function of the interatomic distance, *r*, in Å. The sample label and the weighted residual value, *R*_w_, are displayed to the right in each subplot.

For the pristine materials, the topmost candidate returned by *structureMining* was the TiO_2_-bronze structure (space group *C*2/*m*) (Feist & Davies, 1992 ▸). Fig. 4 ▸(*a*) displays a single-phase fit of the pristine material of batch one, where atomic positions were included in the refinement using space-group constraints and restraints of ±0.05 of the relevant unit-cell side length. A weighted residual value of *R*_w_ = 0.15 was obtained. The coherent spherical domain size was estimated to be 30 (6) Å.

For the pristine material of batch two in Fig. 4 ▸(*c*), the residual from a single-phase PDF refinement using the TiO_2_-bronze phase was extracted and uploaded to the *structureMining* app. The topmost candidate for the residual was the TiO_2_-anatase structure (space group *I*4_1_/*amd*) (Horn *et al.*, 1972 ▸). A weighted residual value of *R*_w_ = 0.17 was obtained, when including atomic positions subject to space-group constraints of the two phases. The estimated weight fractions of the TiO_2_-bronze and anatase phases were 0.85 and 0.15, and the estimated spherical coherent domain sizes were 25 (6) and 40 (40) Å, respectively. The high uncertainty for coherent domain size of the secondary anatase phase should of course be noted. It is common for minor phases to display high uncertainties for scale factors and coherent domain sizes, due to the minor contributions to the total scattering signal. Also, scale factors and coherent domain sizes are often highly correlated, especially for nanosized and minor phases. This should be kept in mind for the estimated weight fractions, as these are based on the scale factors of the refinement.

For the chemically lithiated material of batch one in Fig. 4 ▸(*b*), the topmost candidate was a lithiated version of the TiO_2_-bronze structure (space group *C2*), with the formula LiTi_4_O_8_, *i.e.* Li_0.25_TiO_2_. The fit residual of a single-phase refinement was extracted and uploaded to the *structureMining* app. The topmost candidate was a lithiated version of the TiO_2_-anatase structure with composition Li_7_Ti_8_O_16_, *i.e.* Li_0.875_TiO_2_ (space group 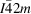). For a two-phase refinement, a weighted residual value of *R*_w_ = 0.16 was obtained together with mass fractions of 0.85 and 0.15 and coherent domain sizes of 26 (7) and 20 (20) Å for the lithiated bronze and anatase phases, respectively. Again, the high uncertainty on the domain size of the minor phase should of course be noted.

For the cathode composite (containing PVDF binder and conductive carbon) and the first *operando* frame in Figs. 4 ▸(*d*) and 4 ▸(*e*), a modified graphite phase (not obtained from the *structureMining* app) was included in addition to the TiO_2_-bronze and anatase phases used for the pristine material. The most evident difference with respect to the PDF of the pristine material in Fig. 4 ▸(*c*) was the additional atomic pair correlations, *e.g.* the C–C correlation at 1.4 Å together with the ‘dilution’ of the TiO_2_ signal, due to the electrode additives. When including atomic position subject to space-group constraints for the TiO_2_-bronze and anatase phases, a weighted residual value of *R*_w_ = 0.25 was obtained. The estimates for the coherent domain sizes of the bronze and anatase phases were similar to those obtained for the pristine material in Fig. 4 ▸(*a*); however, the estimated weight fractions were a little different, as they were estimated as 0.8 and 0.2, respectively, reflecting the level of uncertainty of these estimates.

Fig. 4 ▸(*e*) displays the PDF fit for the first frame of the *operando* experiment. The increase of, *e.g.*, the C–C atomic pair correlation at 1.4 Å reflects the residual signal of the glassy carbon windows of the AMPIX cell, *i.e.* remaining background signal. The modified graphite phase can account for some of the additional residual signal; however, the weighted residual value of *R*_w_ = 0.35 reflects the increased complexity of the system from which the experimental PDF originates. The spherical coherent domain sizes for the TiO_2_-bronze and anatase phases were refined to 30 (12) and 22 (14) Å, respectively, which are a little different from but comparable to the *ex situ* estimates in Figs. 4 ▸(*c*) and 4 ▸(*d*). The estimated weight fractions were 0.8 and 0.2, respectively, which are also comparable to the *ex situ* estimates in Figs. 4 ▸(*c*) and 4 ▸(*d*). In Appendix *C* (supporting information), Fig. C1 displays the PDF fits with contributions from the individual phases and tables with refinement results are provided.

### 3.3. *Operando* PDFs and denoising using PCA

**Figure 5:**
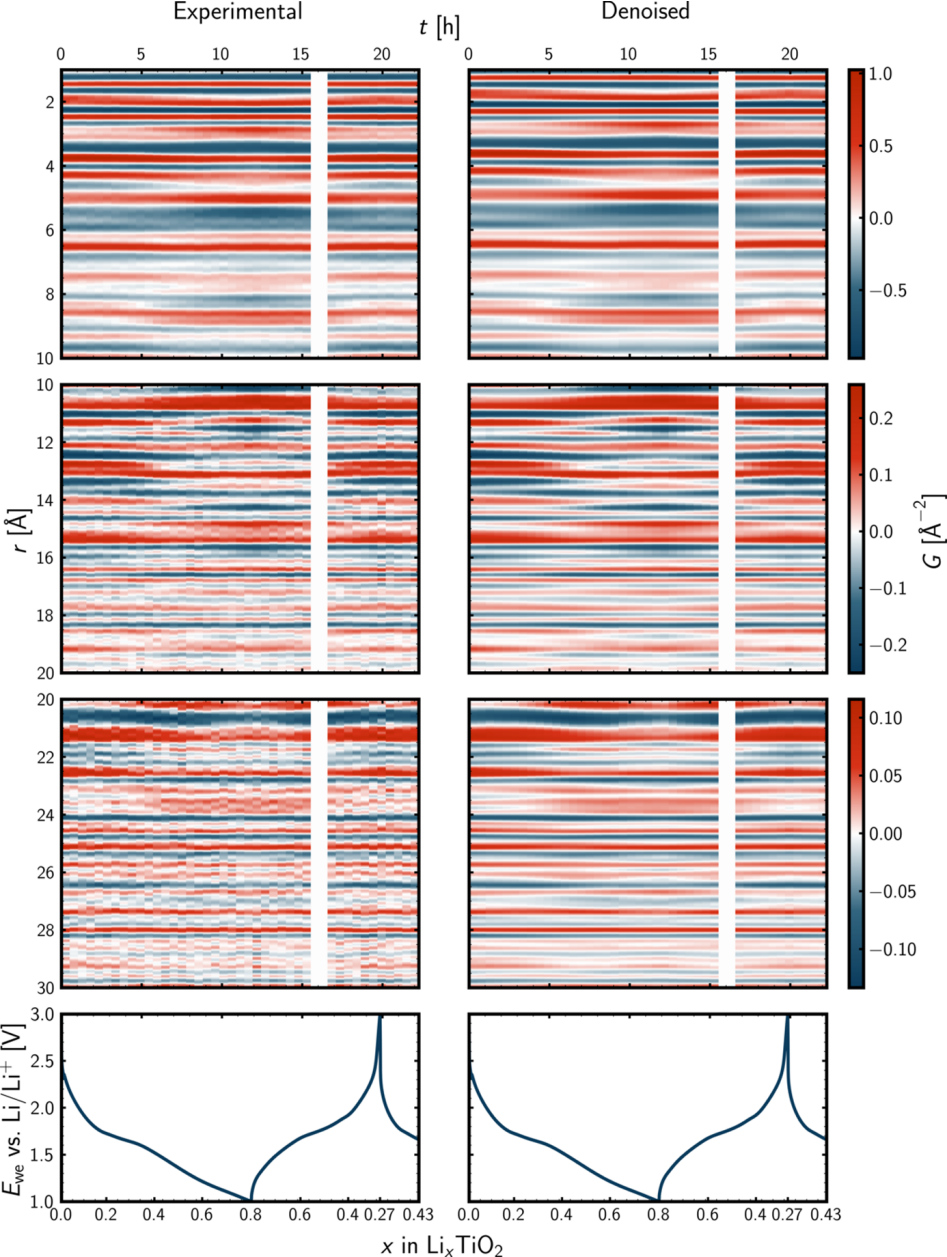
Experimental (left) and denoised (PCA-reconstructed, right) *operando* PDF data together with galvanostatic cycling. The reduced atomic PDF, *G*, as a function of the interatomic distance, *r*, and time, *t*, during the *operando* experiment. Note the different scales for the colour bars of the different *r* ranges. The white columns represent the absence of synchrotron X-rays during the *operando* experiment. For the voltage profile, the electrochemical potential of the working electrode, *E*_we_ versus Li/Li+, is shown as a function of the state of charge, *x*, in Li_*x*_TiO_2_.

For the *operando* PDF data in the left part of Fig. 5 ▸, the signal-to-noise ratio quickly decreases with *r*. Already from around 15 Å, the experimental signal suffers from a relatively low signal-to-noise ratio, resulting from the severe damping of the PDFs originating from both the sample and the instrument. The effect of denoising the experimental *operando* PDF data using PCA is seen in the right part of Fig. 5 ▸. That the PCA serves as a noise filter is clearly seen for the high-*r* region. When denoising, one should be highly aware of what is filtered from the experimental signal. Filtering too much will hamper the signal of interest as not only noise is filtered, which will result in improper interpretation of the data. To arrive at an appropriate level of denoising, PCA was conducted iteratively using one to ten principal components for the matrix decomposition, and the explained variance ratio and its cumulated version were inspected as a function of the number of principal components, as presented in Fig. D1 in Appendix *D* (supporting information). A kink at four components was observed. Therefore, the sklearn.decomposition.PCA class (Pedregosa *et al.*, 2011 ▸) was instantiated with n_components = 4. The difference between the experimental data and the PCA reconstruction, *i.e.* the filtered noise, is presented in Fig. D2 in Appendix *D*. The part of the signal that is captured by the noise filter appears structureless and its level seems to be relatively constant with *r*, as expected from noise of a PDF. Another way of comparing experimental and PCA-reconstructed data is through Pearson correlation analysis, as done in Fig. D3 in Appendix *D*, where it is seen that all the reconstructed PDFs are highly similar to the experimental ones. A direct comparison for the first *operando* PDF is found as Fig. D4 in Appendix *D*, where it is seen that the difference curve behaves as noise, as it should. The denoising using PCA was also done in reciprocal space for the *operandoF*(*Q*) data, which is presented as Figs. D6–D9 in Appendix *D*.

From the low-*r* part of Fig. 5 ▸, it is evident that the very local structure of the electrode material changes very little during the *operando* experiment. This was expected, as we anticipated that the polymorphs to be encountered are all built from TiO_6_ octahedra. Due to the low X-ray scattering power of Li, the appearance of Li correlations in the PDF is not expected. However, the structural response upon Li intercalation resulting in, *e.g.*, an increase in Ti–O distances due to reduction of Ti is expected to be observable, especially for correlations further out in *r*. From visual inspection of the *operando* PDF data and the voltage profile of the galvanostatic cycling in Fig. 5 ▸, the charged state of the electrode at the end of the experiment appears similar to the pristine charged state of the electrode at the beginning. During the initial discharge, some atomic pair correlations fade, *e.g.* around *r* = 9 Å, while others emerge, *e.g.* around *r* = 8 Å. The fading and the emerging of the atomic pair correlations appear to be reversible. During the discharge, the fading and emerging of atomic pair correlations can be linked to the kink of the voltage profile around *x* = 0.4, for *x* in Li_*x*_TiO_2_. During the charge, the related kink is less pronounced, though a delicate kink appears around *x* = 0.6. Similar observations can be made in reciprocal space for the reduced total scattering structure function, *F*(*Q*) (Fig. D6 in Appendix *D*). Thus, from visual inspection of the *operando* data, a reversible two-phase transformation appears to occur. As the discharge capacity of the galvanostatic cycling is larger than the charge capacity, the phase evolution might only be partly reversible.

### 3.4. *similarityMapping* for *operando* PDF data

**Figure 6:**
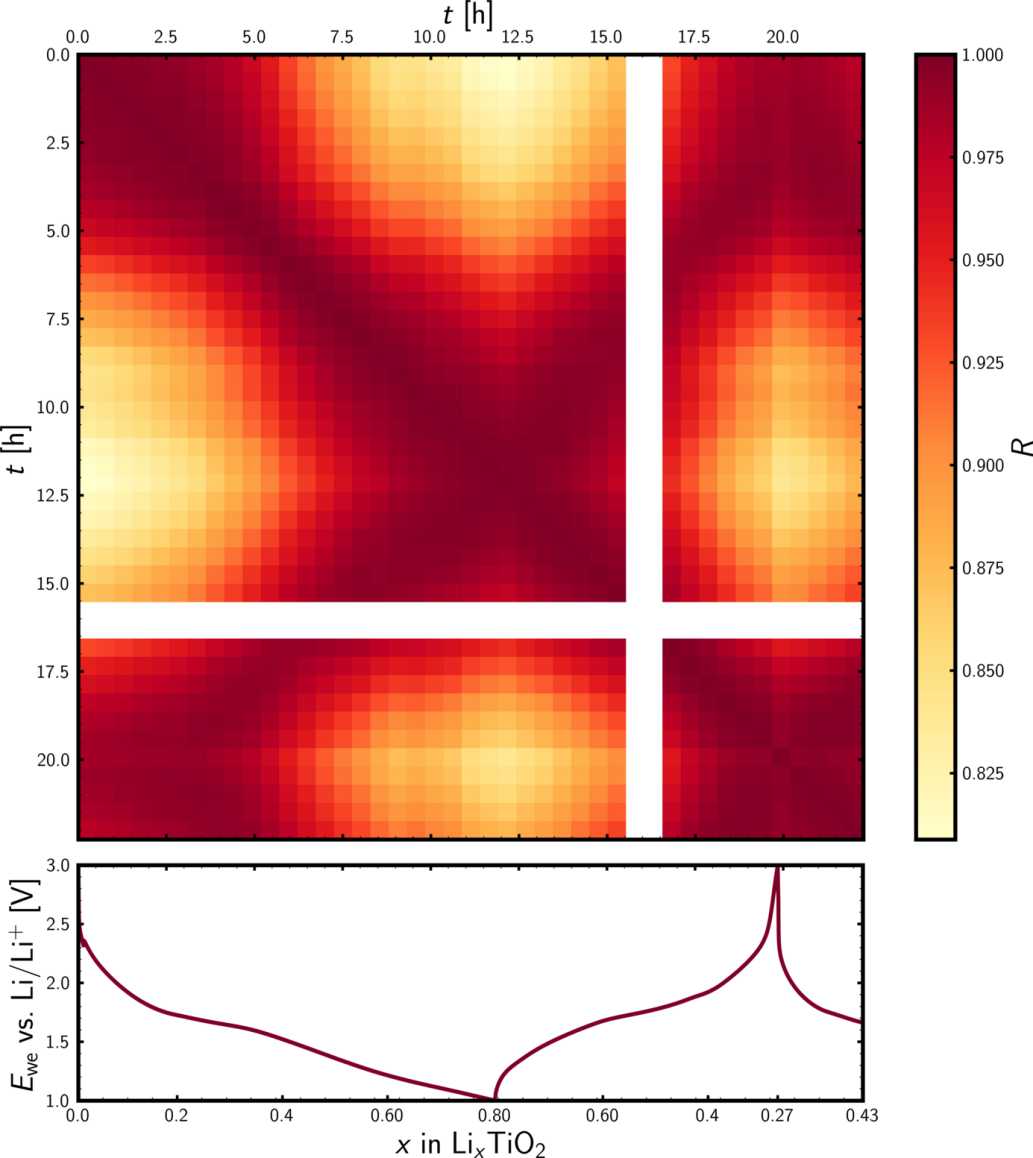
Pearson cross-correlation matrix for the *operando* PDF data. The corresponding time, *t*, is displayed on the axes. The correlation analysis was conducted for the *r* range from 0 to 30 Å. The white columns are due to the absence of synchrotron X-rays during the *operando* experiment. The voltage profile is shown below. The electrochemical potential of the working electrode, *E*_we_ versus Li/Li^+^, is shown as a function of state of charge, *x*, in Li_*x*_TiO_2_.

To probe the similarity between the individual *operando* PDFs, the *similarityMapping* app at *PDFitc* was used. The output of the Pearson correlation analysis is shown in Fig. 6 ▸, which displays the correlation matrix of the *operando* PDFs together with the voltage profile of the galvanostatic cycling. The correlation analysis was conducted for the *r* range from 0 to 30 Å. From the scale of the colour bar, it is seen that all PCCs are above 0.8, indicating no severe structural transformations or reconstructions, which is in agreement with the real-space overview plot in Fig. 5 ▸ and the reciprocal-space overview plot in Fig. D6 in Appendix *D*. The aforementioned domains of the voltage profile and the reversibility identified from Fig. 5 ▸ are also apparent from the correlation matrix. From the correlation matrix, it is seen that the pristine material is highly similar to the Li-poor state upon charge, just as the Li-rich states of the initial discharge are highly similar to the Li-rich state of the charge. Hence, the qualitative interpretations from Fig. 5 ▸ are supported by the quantitative but model-free Pearson correlation analysis conducted using *similarityMapping* at *PDFitc*. Outputs of correlation analyses for various *r* ranges (0–10 Å, 10–20 Å and 20–30 Å) are available in Appendix *E* (Figs. E1–E3, supporting information). The trends of the correlation matrices do not appear to be *r* dependent, though the sensitivity towards similarity is highest for the intermediate *r* range from 10 to 20 Å (Fig. E2). At lower *r* values, the encountered phases are expected to be similar due to the common TiO_6_ octahedral building blocks. At higher *r* ranges, the signal-to-noise ratio is so low that the noise level hampers the sensitivity towards structural dissimilarities, just as atomic pair correlations also are expected to overlap more and more with increasing *r*.

### 3.5. *nmfMapping* for *operando* PDF data

**Figure 7:**
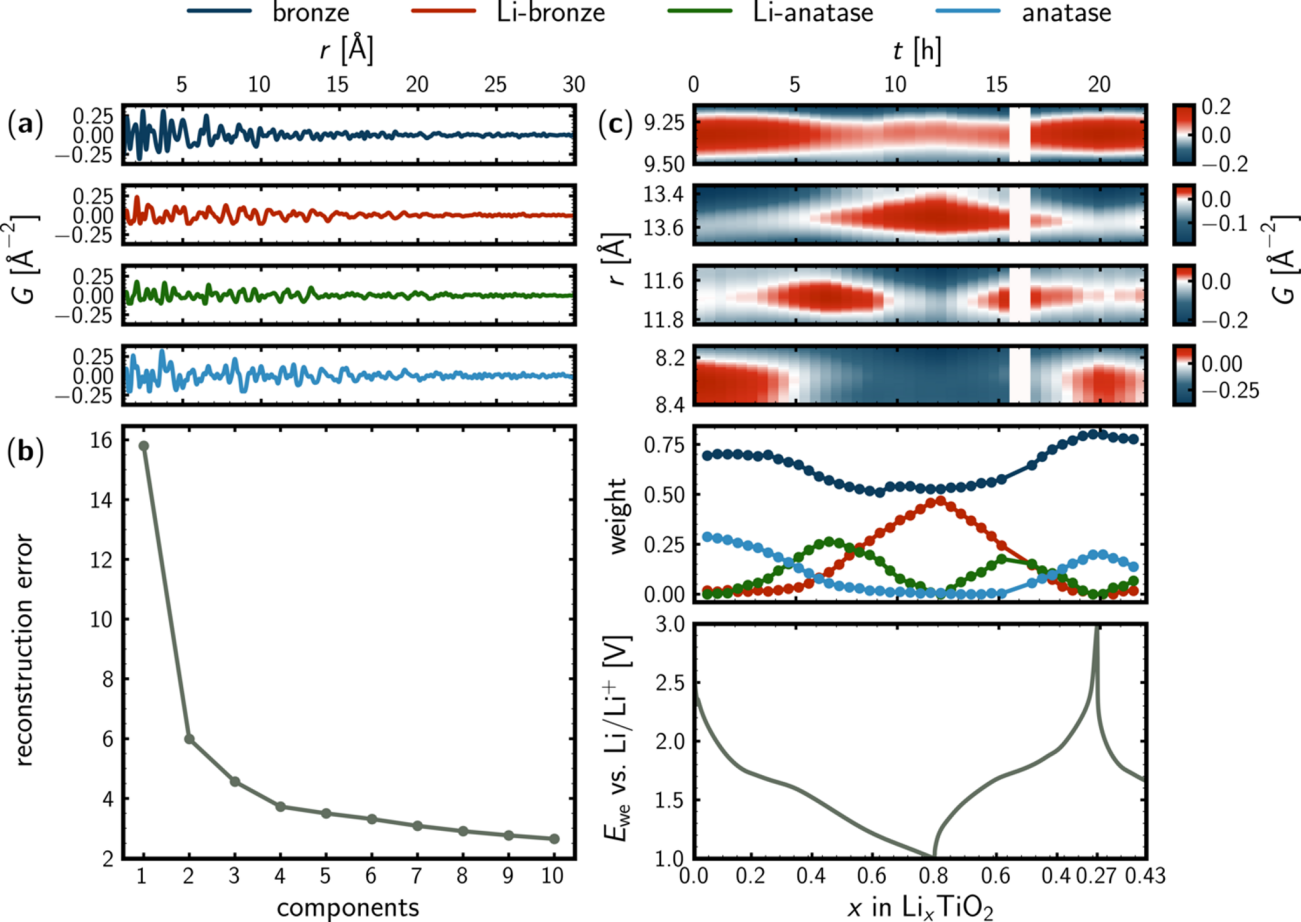
NMF analysis using four components. (*a*) Component PDFs (*G* versus *r*). (*b*) Reconstruction error as a function of the number of components. (*c*) Top: phase-specific atomic pair correlations of the bronze, lithiated bronze, lithiated anatase and anatase phases, from denoised *operando* PDF data. Middle: NMF weights. Bottom: voltage profile. The electrochemical potential is shown as a function of the lithiation degree (state of charge).

The *nmfMapping* app at *PDFitc* was used to obtain insights into the *operando* PDF data through NMF analysis. Running the NMF analysis using four components results in the physically interpretable output in Fig. 7 ▸. The first step in the protocol to arrive at the result in Fig. 7 ▸ was to determine the number of components needed to describe the trends in the *operando* data. This was done by running a query on the *nmfMapping* app, where no number was stated for the number of components to use during the matrix decomposition. Due to the complex and noisy nature of the *operando* PDF data, the default maximum number of components, which is currently ten, was encountered. The reconstruction error as a function of the number of components was used to determine the number of components needed to describe the trends in the data to a sufficient extent, as displayed in Fig. 7 ▸(*b*). A distinct change for the slope of the reconstruction error was observed for two, four and five components. Going from four to five components barely changed the reconstruction error, whereas a constant slope was observed beyond five components. The interpretation was that it was meaningful to include up to four components in the analysis. The constant decrease from five to ten components was expected to arise from the additional degree of freedom that came with every additional component allowed during the matrix decomposition. From here, the NMF analysis was done iteratively, using two, three, four and finally five NMF components. Using up to four NMF components results in output that could be interpreted in a physically meaningful way. Using five NMF components, a physically meaningful interpretation was not possible anymore. That too many components had been used was evident from the behaviour of the NMF weights, just as the component PDFs started to be ‘capped’ at the top and bottom as can be seen in Figs. F7 and F8 in Appendix *F* (supporting information). None of these observations corresponded to physically meaningful behaviour. When using two NMF components (Figs. F1 and F2), the NMF output could be interpreted as one Li-poor and one Li-rich component. That two components were not sufficient could be concluded when bearing in mind the analysis of the *ex situ* PDF data of the 3 nm material of batch two [Fig. 4 ▸(*c*)], where it was evident that two TiO_2_ phases (bronze and anatase) were present initially. Using three NMF components (Figs. F3 and F4), the absence of a second component of the pristine material persisted. Instead, a component emerging and fading during both discharge and charge was realized. The second component for the pristine material appeared when using four NMF components (Fig. 7 ▸ and Fig. F6). There is not a one-to-one correspondence between the NMF components in Fig. 7 ▸ and the individual phase PDFs, as displayed in Fig. F5 in Appendix *F*. The multicomponent nature of the electrochemical cell, which was probed in transmission mode by X-rays, results in lots of scattering contributions other than those originating from the active electrode material of interest. One example is the C–C atomic pair correlation at *r* = 1.4 Å, which is present for all four NMF components and originates from electrode additives as well as the glassy carbon windows of the AMPIX cell. Also, the static NMF components are only allowed to describe the dynamic phase evolutions through linear combinations with the NMF weights (scale factors). Hence, no structural changes are allowed for the NMF components, *e.g.* lattice parameters.

However, the trends in the data described by the NMF weights in Fig. 7 ▸(*c*) line up nicely with the voltage profile. Interestingly, even though four phases seem to be present at intermediate discharge and charge, only two phases are apparent by the end of the discharge and at the end of the charge. The latter feature, together with the overview plot in Fig. 5 ▸ and the *similarityMapping* output in Fig. 6 ▸, indicates the partly reversible nature of the phase behaviour. From the NMF analysis, it appears that two Li-poor phases exist for the pristine material, which are reformed upon charge in a reversible manner. From the *ex situ* PDF analysis (Fig. 4 ▸), the two NMF components are expected to represent the TiO_2_-bronze and TiO_2_-anatase phases. Their reversible formation is also expected from the correlation analysis (Fig. 6 ▸). At deep discharge, the NMF analysis indicates the presence of two NMF components, which are the major pristine component together with another component that emerges during the discharge and fades upon charge. From the *ex situ* PDF analyses (Fig. 4 ▸), the two components are interpreted as representing the Li-poor and Li-rich bronze phases. Finally, the most interesting outcome of the NMF analysis is the NMF component representing an intermediate, which emerges and fades during both the discharge and the charge, where a maximum of the corresponding NMF weight occurs at the kinks of the voltage profile. Its presence also explains the more gradual changes observed for the correlation matrix (Fig. 6 ▸) at intermediate state of charge. From the correlation matrix, it can be seen that the PDF where the weight of the intermediate component is at maximum is more similar to the pristine and charged states than the discharged state, which might indicate a structural similarity to the TiO_2_-anatase phase present at these states of charge. This would be somewhat in line with the *ex situ* PDF analysis of the chemically lithiated material [Fig. 4 ▸(*b*)], which was modelled using lithiated bronze and anatase phases.

Atomic pair correlations that follow the behaviour of the NMF weights have been identified from the denoised *operando* PDF data for each NMF component. These are plotted together with the NMF weights and the voltage profile in Fig. 7 ▸(*c*) to emphasize the physical relevance of the NMF output.

### 3.6. Modelling of *operando* PDF data

**Figure 8:**
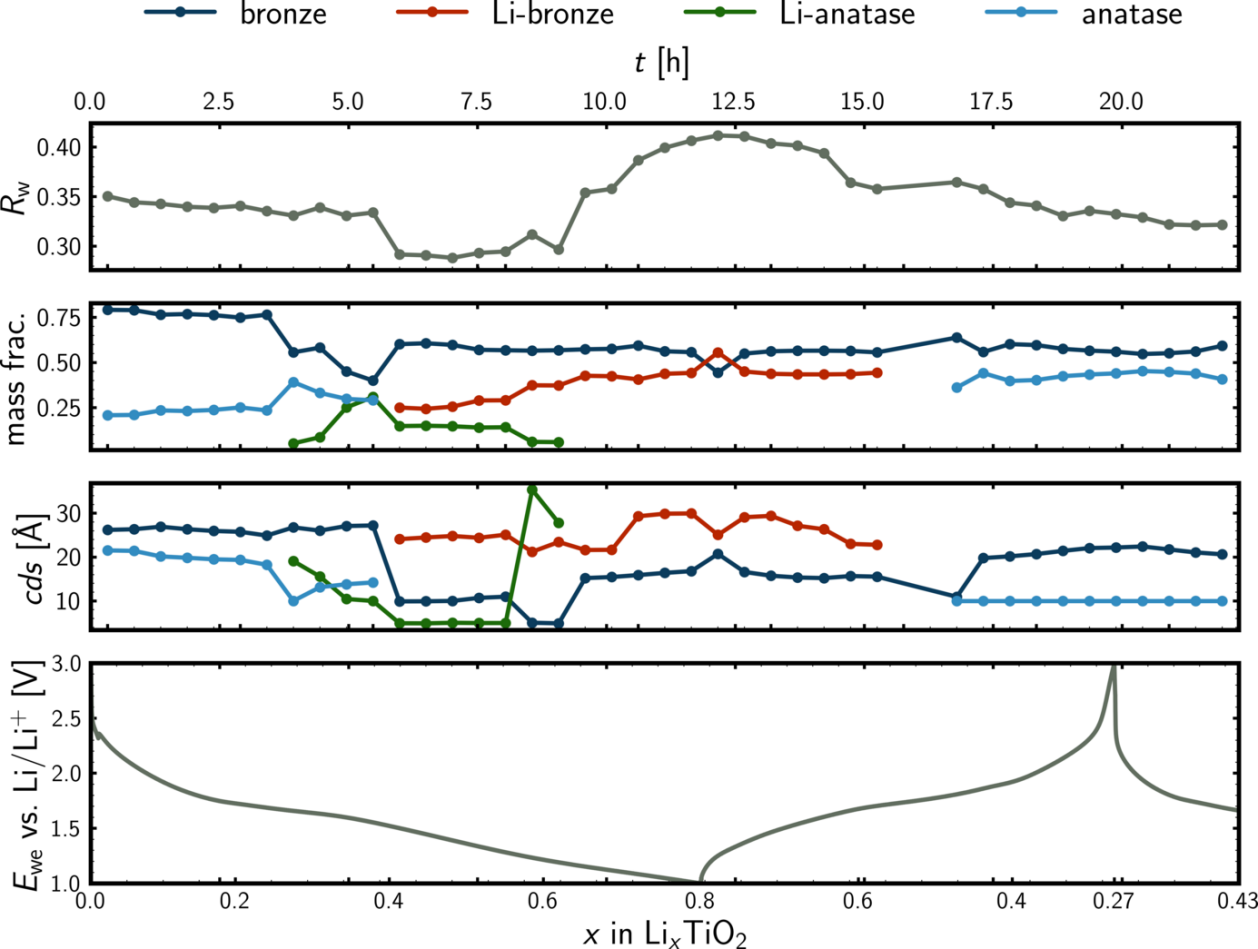
PDF modelling results. The weighted residual values for the PDF refinements, *R*_w_, the mass fractions and the coherent (spherical) domain sizes, cds, for each of the four phases included during the refinement of the *operando* data. The values are plotted for each frame of the *operando* experiment as a function of time, *t*, together with the galvanostatic cycling, showing the working electrode potential, *E*_we_, as a function of the Li content of the positive electrode, *x*, in Li_*x*_TiO_2_.

Fig. 8 ▸ displays refinement results for the *operando* PDF analysis. The nature of the phases appearing during the *operando* analysis was elucidated by the *ex situ* analyses (Fig. 4 ▸). The inclusion of the various phases was greatly guided by the NMF analyses in both real and reciprocal space (Fig. 7 ▸ and Fig. F6 in Appendix *F*). The output of the NMF analyses indicated the presence of four components (phases) during the *operando* experiment on the TiO_2_ nanocrystals. Because of the similarity of the TiO_2_ and Li_*x*_TiO_2_ polymorphs from the Pearson correlation analysis in Fig. 6 ▸, together with the rather limited range of data to refine (*r* range from 1.2 to 20 Å), due to sample and instrumental damping, the PDF analysis was highly challenging. The weighted residual values ranging from a little below 0.3 to a little above 0.4 in Fig. 8 ▸ indicate reasonable fits, bearing in mind the *operando* nature of the PDF data. The reversible nature of the phase evolution was reflected by comparable descriptors of the pristine state and charged states, though the coherent domain sizes are observed to decrease a little for the charged states compared with the pristine state, which probably also explains the discrepancy for the estimated weight fractions. In particular, the rather low coherent domain size estimated for the anatase phase, in light blue, is expected to hamper the weight fraction estimates. In Figs. G1–G4 in Appendix *G* (supporting information), PDF fits with individual phase contributions are shown for the pristine material, the midpoint of the initial discharge, the end of the initial discharge and the end of the charge.

A clear challenge for the *operando* analysis was the detection limits, especially for the regions where three- or four-phase systems were expected from the NMF analyses. For instance, this was seen during the charge, where it was not possible to include the intermediate Li_*x*_TiO_2_-anatase phase, even though it was expected to be present, both from the NMF analyses and also from inspection of the *operando* data, as highlighted in Fig. 7 ▸. Of course, the absence of minor phases in the structural modelling is to be kept in mind when evaluating the modelling results, as is always the case for incomplete (*i.e.* all) models. However, being able to compare such modelling results with the results of other types of analyses, *e.g.* Pearson correlation analysis and NMF analysis, is of immense value, as this provides a measure of trustworthiness, increasing the value of the modelling results significantly. Refined unit-cell parameters for each of the phases, including the modified graphite phase, can be found as Figs. G5–G9 in Appendix *G*.

From the NMF analysis of the *operando* data in Fig. 8 ▸, the TiO_2_-anatase (light blue) phase appears to transform before the TiO_2_-bronze phase (navy) upon Li-ion intercalation during the discharge. The TiO_2_-anatase phase transforms into a lithiated analogue, Li_*x*_TiO_2_-anatase (green), which is an intermediate, as the phase is absent at deep discharge: 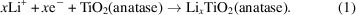
The phase transition of TiO_2_-anatase into Li_0.5_TiO_2_-anatase has previously been reported (Murphy *et al.*, 1983 ▸; Lafont *et al.*, 2010 ▸). When the formation of the intermediate Li_*x*_TiO_2_-anatase is complete, around *x* = 0.4 in Li_*x*_TiO_2_, the lithiated bronze phase Li_*x*_TiO_2_-bronze (red) forms from the TiO_2_-bronze as well as from the Li_*x*_TiO_2_-anatase:
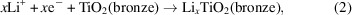

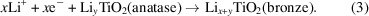
At deep discharge, a biphasic mixture of TiO_2_-bronze and Li_*x*_TiO_2_-bronze is present: due to reactions (2)–(3).

Upon recharge, the reverse behaviour is observed and the phase behaviour appears to be somewhat reversible, as also indicated by Pearson correlation analysis in Fig. 6 ▸:
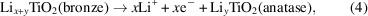

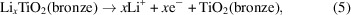

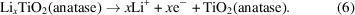
A possible interpretation of these observations would be that the minor and more disordered TiO_2_-anatase phase is related to the surface of the nanocrystals. The surface would be lithiated before the ‘bulk’ of the nanocrystals, such that the lithiated anatase phase forms before the lithiated bronze phase. Another possibility would be individual bronze and anatase nanoparticles, where the anatase particles are lithiated before the bronze particles.

The transformation of Li_*x*_TiO_2_-anatase to Li_*x*_TiO_2_-bronze at *x* ≳ 0.4 may be enabled by the small domain size of the anatase phase, as the anatase phase usually is not lithiated beyond *x* ∼ 0.5 (Murphy *et al.*, 1983 ▸; Lafont *et al.*, 2010 ▸). However, whether this would also occur in single-phase anatase materials remains to be investigated.

## 4. Conclusions

Our work demonstrates various approaches to obtain insights into the atomic structure, phase contents and structural tranformations of nanocrystalline functional materials through *ex situ* and time-series (*operando* and *in situ*) PDF analysis. The toolbox has been demonstrated for the case of TiO_2_-bronze nanocrystals, which were studied during galvanostatic cycling when incorporated into the positive electrode of a rechargeable Li-ion battery. The nanosize and multiphase nature of the system result in highly complex *ex situ* and *operando* PDF data. Use of the *structureMining* app at *PDFitc* significantly sped up the screening of both primary and secondary phases of *ex situ* PDF data. The visualization of the *operando* data was greatly improved through denoising using PCA, easing qualitative data interpretation. The *similarity­Mapping* app at *PDFitc* was used for Pearson correlation analysis of the *operando* data to identify the nature and extent of phase transformations. The *nmfMapping* app at *PDFitc* was used for NMF analysis of the *operando* data and provided invaluable insights into the phase evolution of the *operando* data. All of these model-free analysis tools made it possible to model the *operando* PDF data for this highly complex nanocrystalline system, which involves two pristine nanocrystalline phases of TiO_2_-bronze and anatase phases, together with nanocrystalline lithiated bronze and anatase phases. The structural evolution appears to be reversible, though the charge capacity is lower than the inital discharge capacity, indicating some irreversibility of the system. Without using the various tools of the toolbox presented herein, the structural modelling would have been immensely complicated to complete. However, careful use, *e.g.*, through use of chemical intuition, enabled the full analysis presented. All of the model-free analyses are fairly quick to conduct and their unbiased model-free nature means that they may reveal unexpected features of the data. Taking this into account, the limited amount of time needed to conduct these analyses is well spent. We are fully convinced that the toolbox presented herein will be of great use to many others dealing with *ex situ* and time-series (*operando* and *in situ*) PDF data.

## 5. Related literature

The following additional literature is cited in the supporting information: Aykol *et al.* (2018 ▸), Jain *et al.* (2013 ▸), Kataoka *et al.* (2011 ▸), Khitrova *et al.* (1977 ▸), Ouhenia *et al.* (2006 ▸), Parker (1924 ▸) and Wyckoff (1963 ▸).

## Supplementary Material

Appendix A. Rietveld anaylsis of ex situ data for batch 1. DOI: 10.1107/S1600576724005624/vb5071sup1.pdf

Appendix B. structureMining outputs. DOI: 10.1107/S1600576724005624/vb5071sup2.pdf

Appendix C. Ex situ PDF refinements. DOI: 10.1107/S1600576724005624/vb5071sup3.pdf

Appendix D. Noise-filtering using principal component analysis (PCA). DOI: 10.1107/S1600576724005624/vb5071sup4.pdf

Appendix E. similarityMapping: Pearson correlation analysis for operando PDF data. DOI: 10.1107/S1600576724005624/vb5071sup5.pdf

Appendix F. nmfMapping: non-negative matrix factorization for operando data. DOI: 10.1107/S1600576724005624/vb5071sup6.pdf

Appendix G. Operando PDF modelling. DOI: 10.1107/S1600576724005624/vb5071sup7.pdf
