# Supplementary material for: Operando pair distribution function analysis of nanocrystalline functional materials: the case of TiO2-bronze nanocrystals in Li-ion battery electrodes
Source: J Appl Crystallogr. 2024 Jul 29;57(Pt 4):1171–83. doi: 10.1107/S1600576724005624 (PMC11299615; doi:10.1107/S1600576724005624)
Supplement: Supplementary file 1 [file j-57-01171-sup1.pdf]

JOURNAL OF
APPLIED
CRYSTALLOGRAPHY

Volume 57 (2024)

Supporting information for article:

Operando pair distribution function analysis of nanocrystalline functional materials: the case of TiO2-bronze nanocrystals in Li-ion battery electrodes

Martin A. Karlsen, Jonas Billet, Songsheng Tao, Isabel Van Driessche, Simon J. L. Billinge and Dorthe B. Ravnsbæk

Appendix A
Rietveld analysis of *ex situ* data for batch one

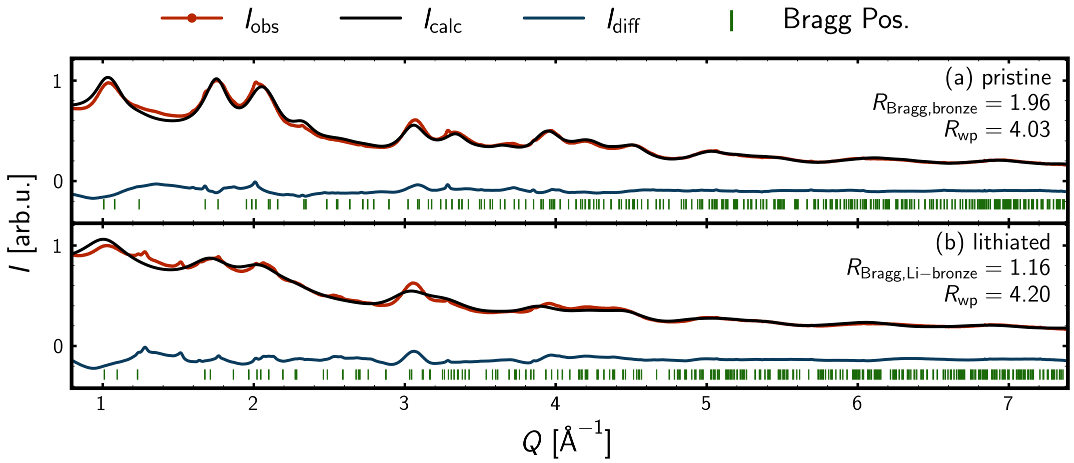

Fig. A1. The observed intensity, I_{obs}, is displayed as red dotted lines, the calculated intensity, I_{calc}, is shown as a black line, and the difference between the observed and calculated intensities, I_{diff}, is displayed as a blue line. The Bragg positions of the phases included in the refinements are indicate by vertical green lines. The Bragg residual values, R_{Bragg}, and the weighted profile residuals, R_{wp}, are shown to the right in each fit. (a) The single-phase fit of the pristine material. (b) The single-phase fit of the chemically lithiated material.

Fig. A1 displays the fits from Rietveld analysis of the *ex situ* data for the pristine and chemically lithiated material of batch one. For the pristine 3 nm material in Fig. A1a, a crystallite size of 3.36 nm was estimated, well in line with expected size of 3 nm. Peaks that could be described by the TiO_2-bronze phase were observed, indicating that at least one secondary phase was present. The fit from the Rietveld analysis of the chemically lithiated material is shown in Fig. A1b. A single phase of lithiated bronze was used. The estimated crystallite size was 1.51 nm. Peaks that could not be described by the $Li_{0.5}TiO_2$ phase were observed, indicating that at least one secondary phase was present. Due to the very broad nature of the features not described by the

TiO_2-bronze and $Li_{0.5}TiO_2$ phases, it was not attempted to include and a secondary phase in the Rietveld analyses of the materials. Comparing the data of the chemically lithiated material to the data of the pristine counterpart, additional peak broadening is observed for the chemically lithiated material, indicative of domain size decrease and/or increase in microstrain upon chemical lithiation.
