# Supplementary material for: Operando pair distribution function analysis of nanocrystalline functional materials: the case of TiO2-bronze nanocrystals in Li-ion battery electrodes
Source: J Appl Crystallogr. 2024 Jul 29;57(Pt 4):1171–83. doi: 10.1107/S1600576724005624 (PMC11299615; doi:10.1107/S1600576724005624)
Supplement: Supplementary file 2 [file j-57-01171-sup2.pdf]

JOURNAL OF APPLIED CRYSTALLOGRAPHY

Volume 57 (2024)

Supporting information for article:

Operando pair distribution function analysis of nanocrystalline functional materials: the case of TiO2-bronze nanocrystals in Li-ion battery electrodes

Martin A. Karlsen, Jonas Billet, Songsheng Tao, Isabel Van Driessche, Simon J. L. Billinge and Dorthe B. Ravnsbæk

Appendix B
structureMining outputs

Batch 1

3 nm: pristine

Table B1. STRUCTUREMINING output for the *ex situ* PDF data of the batch one pristine material, when setting the composition to TiO_2. The weighted residual, R_w, the space group, the database from which the CIF originated, the database ID, and the reference in which the CIF was published.

R_w	Space group	Database	ID	Reference
0.26	$C2/m$	MPD	554278	(Feist & Davies, 1992)
0.61	$I4/m$	MPD	1101022	(Jain *et al.*, 2013)
0.74	$C2/m$	COD	1528778	(Ouhenia *et al.*, 2006)
0.74	$P1$	MPD	1245308	(Aykol *et al.*, 2018)
0.78	$P1$	MPD	1245134	(Aykol *et al.*, 2018)

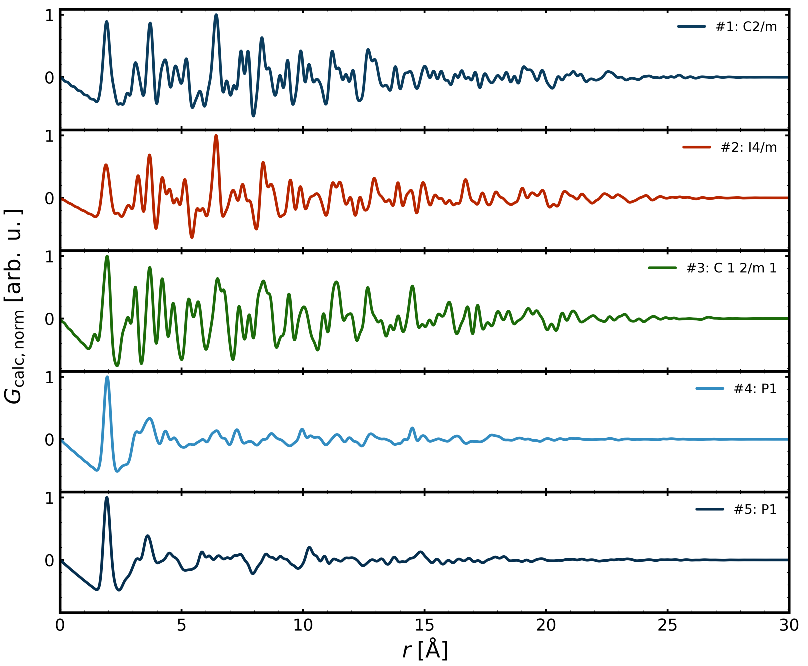

Fig. B1. Normalized calculated PDFs for the entries in Table B1.

3 nm: lithiated

Table B2. STRUCTUREMINING output for the *ex situ* PDF data of the batch one chemically lithiated material, when setting the composition to Li-Ti-O. The weighted residual, R_w, the chemical formula, the space group, the database from which the CIF originated, the database ID, and the reference in which the CIF was published.

R_w	Formula	Space group	Database	ID	Reference
0.37	$LiTi_4O_8$	$C2$	MPD	554278	(Jain *et al.*, 2013)
0.46	$Li_2Ti_6O_{13}$	$C2/m$	COD	7206075	(Kataoka *et al.*, 2011)
0.63	$Li_8Ti_2O_7$	$P2_1/c$	MPD	1526931	(Jain *et al.*, 2013)
0.63	$LiTi_8O_{13}$	$R\bar{3}$	MPD	2310710	(Jain *et al.*, 2013)
0.71	$LiTi_2O_4$	$C2/m$	MPD	9008213	(Armstrong *et al.*, 2010)

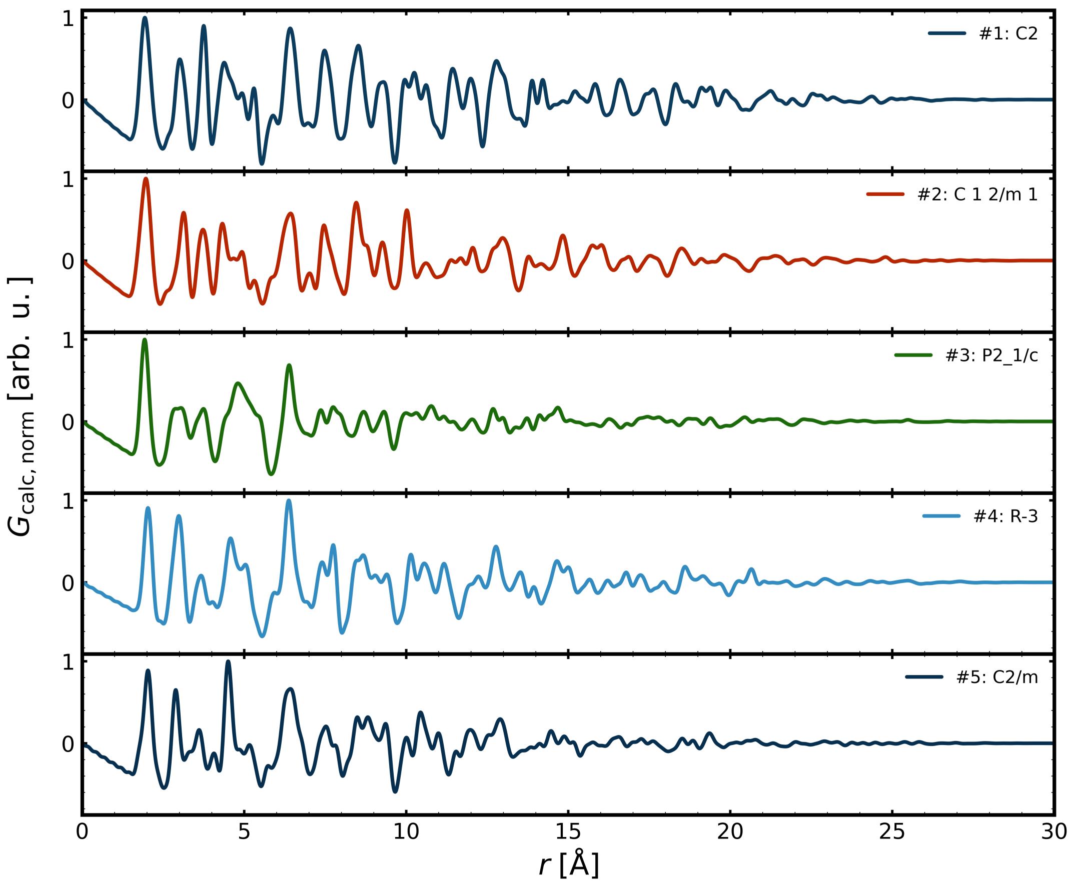

Fig. B2. Normalized calculated PDFs for the entries in Table B2.

3 nm: lithiated, residual

Table B3. STRUCTUREMINING output for the DIFFPY-CMI fit residual for the *ex situ* PDF data of the residual of the batch one chemically lithiated material, when setting the composition to Li-Ti-O. The weighted residual, R_w, the chemical formula, the space group, the database from which the CIF originated, the database ID, and the reference in which the CIF was published.

R_w	Formula	Space group	Database	ID	Reference
0.89	$Li_7Ti_{16}O_{32}$	$I\bar{4}2m$	MPD	530141	(Jain *et al.*, 2013)
0.89	$LiTi_3O_4$	$Cmmm$	MPD	867744	(Jain *et al.*, 2013)
0.90	$Li_4Ti_3O_8$	$C2/m$	MPD	755266	(Jain *et al.*, 2013)
0.90	$LiTi_8O_{16}$	$P\bar{4}m2$	MPD	1222545	(Jain *et al.*, 2013)
0.90	Li_2TiO_3	$P\bar{1}$	MPD	760017	(Jain *et al.*, 2013)

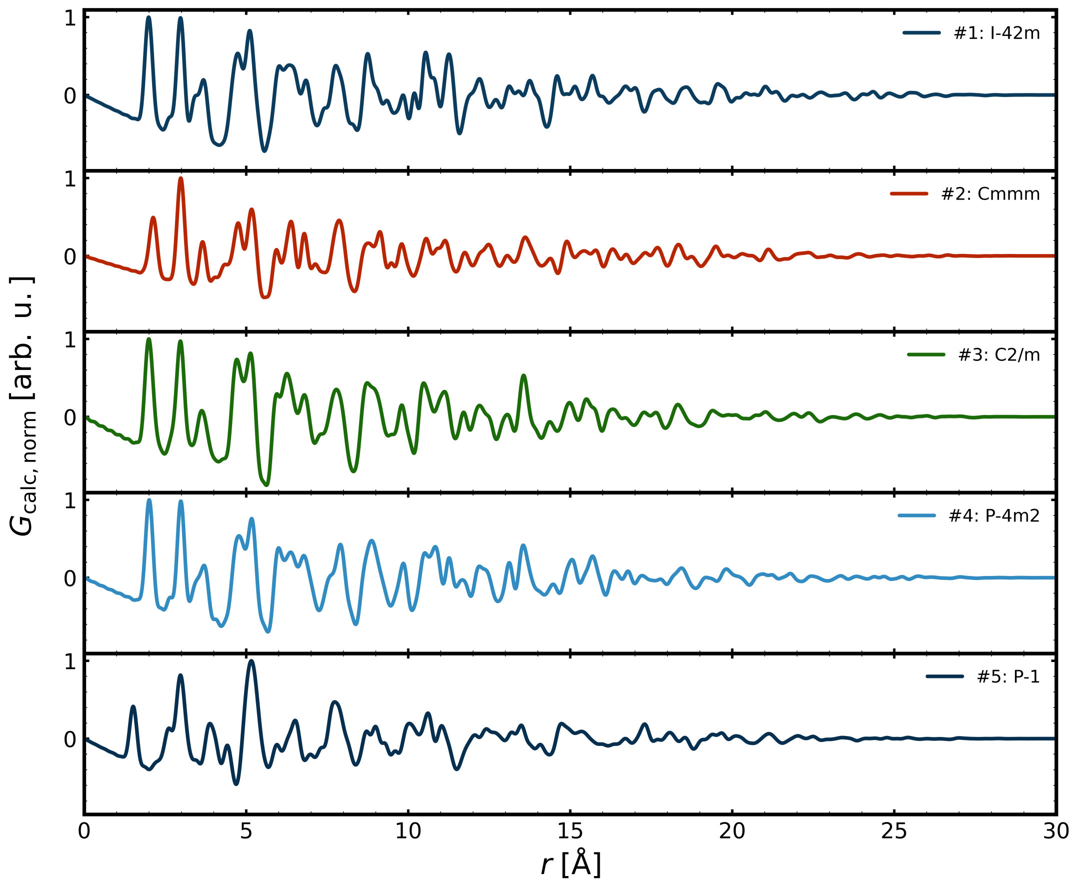

Fig. B3. Normalized calculated PDFs for the entries in Table B3.

Batch 2

3 nm: pristine

Table B4. STRUCTUREMINING output for the *ex situ* PDF data of the batch two pristine material, when setting the composition to TiO_2. The weighted residual, R_w, the space group, the database from which the CIF originated, the database ID, and the reference in which the CIF was published.

R_w	Space group	Database	ID	Reference
0.37	$C2/m$	MPD	554278	(Feist & Davies, 1992)
0.71	$I4/m$	MPD	1101022	(Jain *et al.*, 2013)
0.72	$C2/m$	COD	1528778	(Ouhenia *et al.*, 2006)
0.74	$P1$	MPD	1245308	(Aykol *et al.*, 2018)
0.75	$Pbca$	COD	8104269	(Pauling & Sturdivant, 1928)

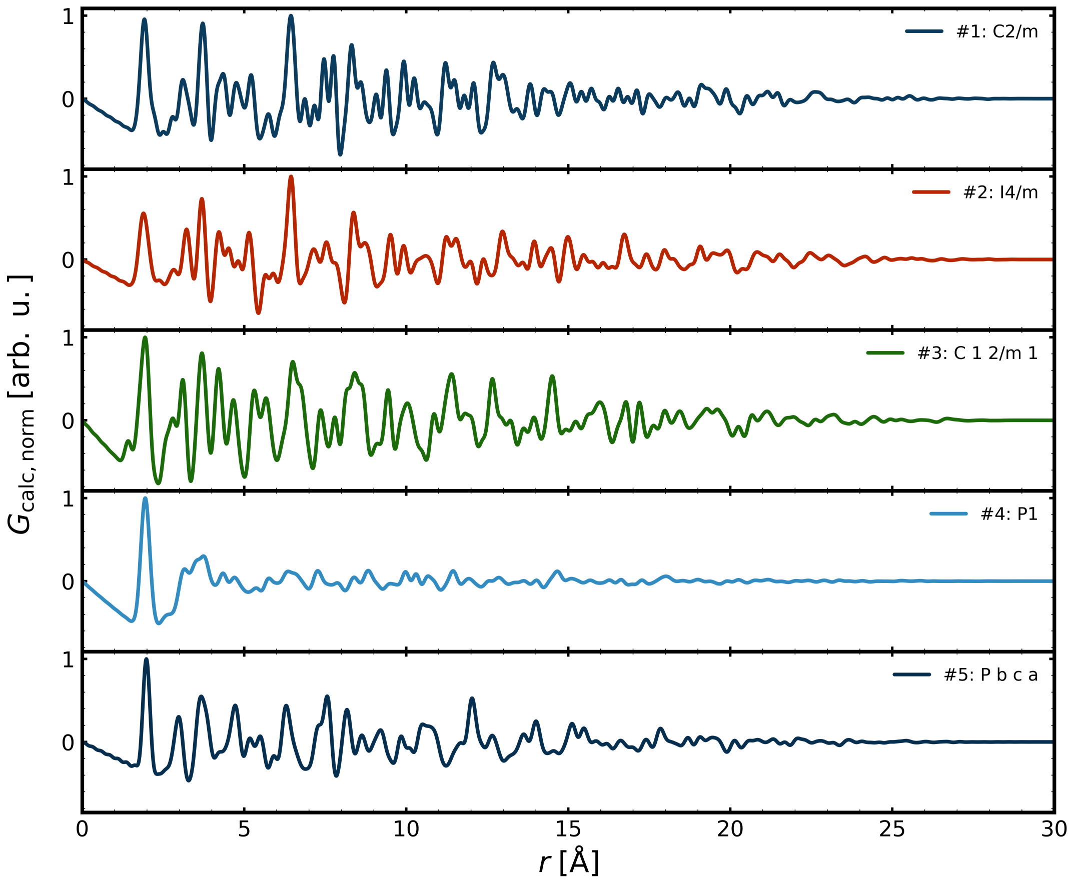

Fig. B4. Normalized calculated PDFs for the entries in Table B4.

3 nm: pristine, residual

Table B5. STRUCTUREMINING output for the DIFFPY-CMI fit residual for the *ex situ* PDF data of the residual of the batch two pristine material, when setting the composition to TiO_2. The weighted residual, R_w, the space group, the database from which the CIF originated, the database ID, and the reference in which the CIF was published.

R_w	Space group	Database	ID	Reference
0.88	$I4_1/amd$	COD	1530151	(Khitrova *et al.*, 1977)
0.90	$I4_1/amd$	COD	1010942	(Parker, 1924)
0.90	$I4_1/amd$	MPD	390	(Jain *et al.*, 2013)
0.90	$I4_1/amd$	COD	9009086	(Wyckoff, 1963)
0.90	$I4_1/amd$	COD	9008216	(Horn *et al.*, 1972)

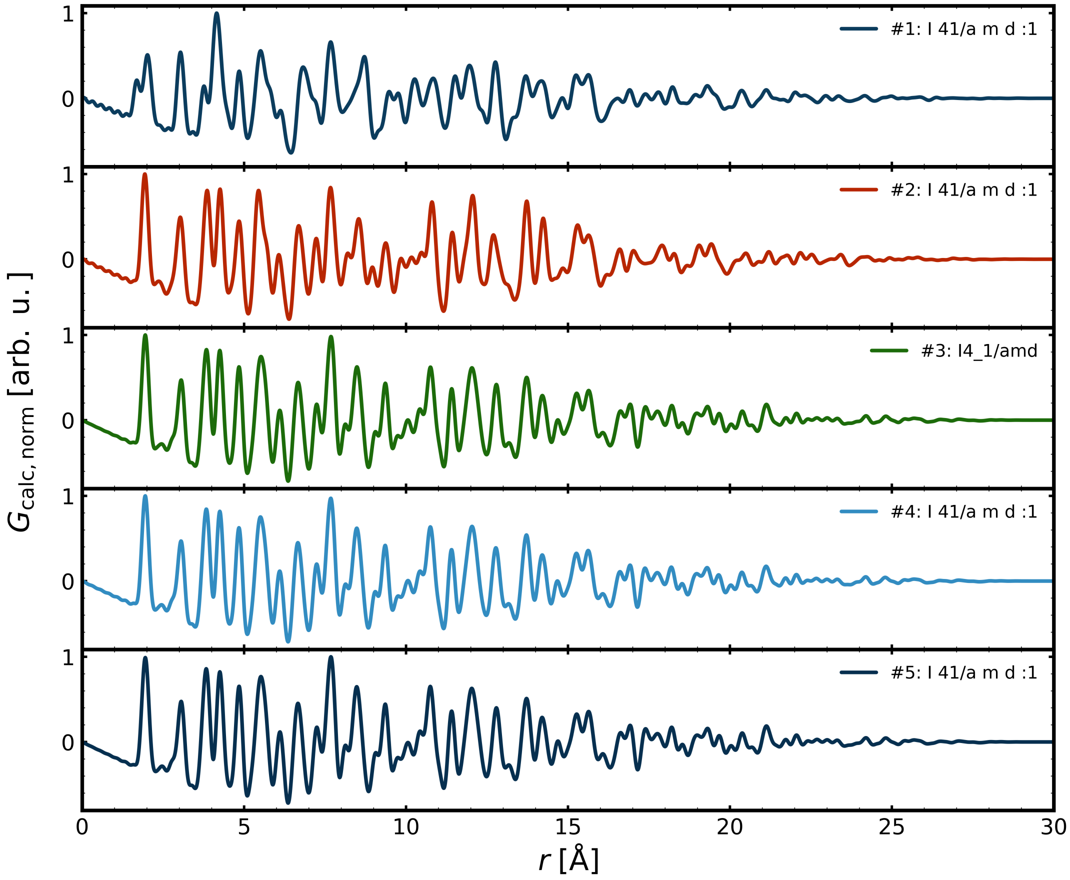

Fig. B5. Normalized calculated PDFs for the entries in Table B5.
