# Supplementary material for: Operando pair distribution function analysis of nanocrystalline functional materials: the case of TiO2-bronze nanocrystals in Li-ion battery electrodes
Source: J Appl Crystallogr. 2024 Jul 29;57(Pt 4):1171–83. doi: 10.1107/S1600576724005624 (PMC11299615; doi:10.1107/S1600576724005624)
Supplement: Supplementary file 3 [file j-57-01171-sup3.pdf]

JOURNAL OF APPLIED CRYSTALLOGRAPHY

Volume 57 (2024)

Supporting information for article:

Operando pair distribution function analysis of nanocrystalline functional materials: the case of TiO2-bronze nanocrystals in Li-ion battery electrodes

Martin A. Karlsen, Jonas Billet, Songsheng Tao, Isabel Van Driessche, Simon J. L. Billinge and Dorthe B. Ravnsbæk

Appendix C
Ex situ PDF refinements

PDF fits w. phase contributions

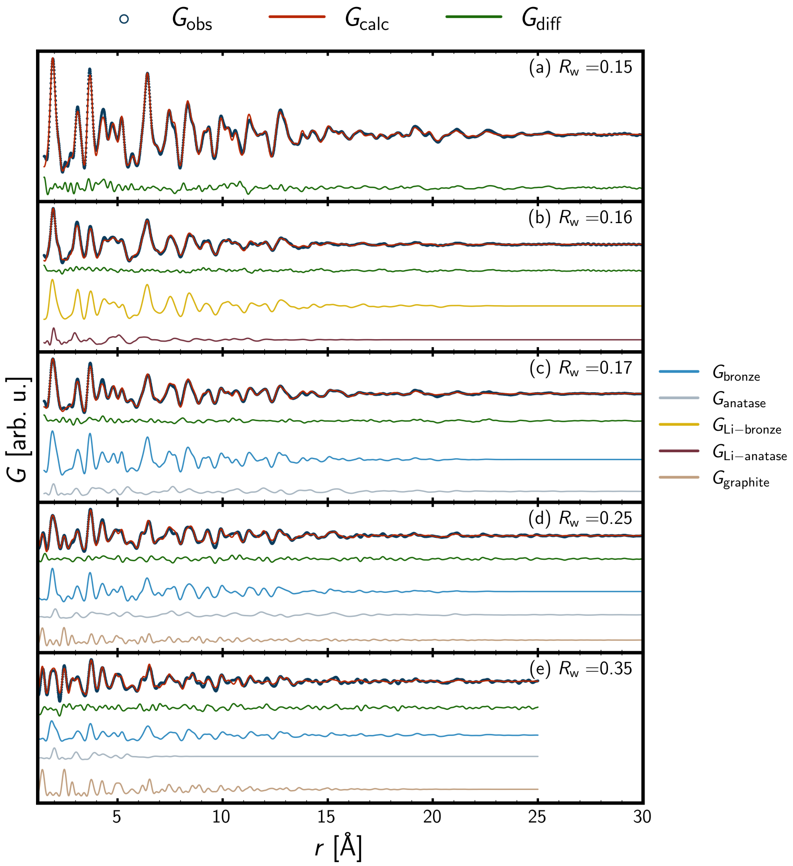

Fig. C1. *Ex situ* PDF refinements. The reduced atomic pair distribution function, G, in arbitrary units, arb. u., is shown as a function of the interatomic distance, r, in Ångström, Å. The observed PDF, $G_{\rm obs}$, is shown as blue circles, the calculated PDF, $G_{\rm calc}$, is shown as a red line, and the difference between the observed and calculated PDFs, $G_{\rm diff}$, is shown as a green line. Contributions for individual phases are shown as cyan (bronze), grey (anatase), yellow (lithiated bronze), maroon (lithiated anatase), and beige (modified graphite) lines. The weighted residual value, $R_{\rm w}$, is displayed to the right in each subplot. (a) pristine, batch 1, (b) lithiated, batch 1, (c) pristine, batch 2, (d) cathode mix, batch 2, and (e) operando cell, batch 2, first frame.

Batch one: pristine material

Table C1. Results from one-phase refinement of *ex situ* PDF data for the pristine material of batch one.

**Variable [unit]**	**Value ± esd**
Scale	0.35 ± 0.06
a [Å]	12.17 ± 0.06
b [Å]	3.74 ± 0.02
c [Å]	6.49 ± 0.04
β [°]	107.1 ± 0.5
$u_{\mathrm{iso,Ti}}$ $\left[\mathrm{Å}^2\right]$	0.006 ± 0.002
$u_{\mathrm{iso,O}}$ $\left[\mathrm{Å}^2\right]$	0.015 ± 0.009
δ_2 $\left[\mathrm{Å}^{-2}\right]$	2.4 ± 1.1
cds [Å]	30 ± 6
$x_{\mathrm{Ti,1}}$ $[a]$	0.101 ± 0.004
$x_{\mathrm{Ti,2}}$ $[a]$	0.197 ± 0.004
$x_{\mathrm{O,1}}$ $[a]$	0.06 ± 0.02
$x_{\mathrm{O,2}}$ $[a]$	0.138 ± 0.009
$x_{\mathrm{O,3}}$ $[a]$	0.132 ± 0.012
$x_{\mathrm{O,4}}$ $[a]$	0.240 ± 0.015
$z_{\mathrm{Ti,1}}$ $[c]$	0.707 ± 0.007
$z_{\mathrm{Ti,2}}$ $[c]$	0.288 ± 0.005
$z_{\mathrm{O,1}}$ $[c]$	0.37 ± 0.02
$z_{\mathrm{O,2}}$ $[c]$	0.011 ± 0.001
$z_{\mathrm{O,3}}$ $[c]$	0.71 ± 0.02
$z_{\mathrm{O,4}}$ $[c]$	0.35 ± 0.02
R_{w}	0.15

Batch one: lithiated material (two Li_xTiO_2 phases)

Li_xTiO_2-bronze

Table C2. Results for the lithiated bronze phase from two-phase refinement of *ex situ* PDF data for the chemically lithiated material of batch one. Please see Table C3 for results of the lithiated anatase phase and the weighted residual.

Variable [unit]	Value ± esd
Scale factor	0.32 ± 0.09
a [Å]	12.3 ± 0.1
b [Å]	3.79 ± 0.03
c [Å]	6.45 ± 0.08
β [°]	107 ± 1
$u_{\mathrm{iso,Ti}}$ $\left[\text{Å}^2\right]$	0.009 ± 0.005
$u_{\mathrm{iso,O}}$ $\left[\text{Å}^2\right]$	0.02 ± 0.03
δ_2 $\left[\text{Å}^{-2}\right]$	2 ± 2
cds [Å]	26 ± 7
$x_{\mathrm{Ti,1}}$ $[a]$	0.103 ± 0.007
$x_{\mathrm{Ti,2}}$ $[a]$	0.201 ± 0.007
$x_{\mathrm{O,1}}$ $[a]$	0.06 ± 0.04
$x_{\mathrm{O,2}}$ $[a]$	0.11 ± 0.02
$x_{\mathrm{O,3}}$ $[a]$	0.16 ± 0.04
$x_{\mathrm{O,4}}$ $[a]$	0.25 ± 0.02
$y_{\mathrm{Ti,1}}$ $[a]$	0.54 ± 0.08
$y_{\mathrm{Ti,2}}$ $[a]$	0.54 ± 0.10
$y_{\mathrm{O,1}}$ $[a]$	0.5355 ± 0.0010
$y_{\mathrm{O,2}}$ $[a]$	0.050 ± 0.002
$y_{\mathrm{O,3}}$ $[a]$	0.4458 ± 0.0010
$y_{\mathrm{O,4}}$ $[a]$	0.02 ± 0.1
$z_{\mathrm{Ti,1}}$ $[c]$	0.708 ± 0.007
$z_{\mathrm{Ti,2}}$ $[c]$	0.287 ± 0.006
$z_{\mathrm{O,1}}$ $[c]$	0.35 ± 0.03
$z_{\mathrm{O,2}}$ $[c]$	0.01 ± 0.03
$z_{\mathrm{O,3}}$ $[c]$	0.17 ± 0.03
$z_{\mathrm{O,4}}$ $[c]$	0.36 ± 0.03
Weight frac.	0.85

Li_xTiO_2-anatase

Table C3. Results for the lithiated anatase phase from two-phase refinement of *ex situ* PDF data for the chemically lithiated material of batch one. Please see Table C2 for results of the lithiated bronze phase.

**Variable [unit]**	**Value ± esd**
Scale factor	0.06 ± 0.06
a [Å]	8.2 ± 0.2
c [Å]	17.7 ± 0.6
$u_{\mathrm{iso,Ti}}$ $\left[\text{Å}^2\right]$	0.01 ± 0.03
$u_{\mathrm{iso,O}}$ $\left[\text{Å}^2\right]$	0.005 ± 0.031
δ_2 $\left[\text{Å}^{-2}\right]$	3.8 ± 0.2
cds [Å]	20 ± 19
Weight frac.	0.15
R_{w}	0.16

Batch two: pristine (two TiO_2 phases)

TiO_2-bronze

Table C4. Results for the bronze phase from two-phase refinement of *ex situ* PDF data for the pristine sample of batch two. Please see Table C5 for results of the anatase phase.

**Variable [unit]**	**Value ± esd**
Scale	0.32 ± 0.07
a [Å]	12.14 ± 0.08
b [Å]	3.75 ± 0.02
c [Å]	6.51 ± 0.05
β [°]	107.0 ± 0.7
$u_{\mathrm{iso,Ti}}$ $\left[\mathrm{Å}^2\right]$	0.005 ± 0.002
$u_{\mathrm{iso,O}}$ $\left[\mathrm{Å}^2\right]$	0.012 ± 0.009
δ_2 $\left[\mathrm{Å}^{-2}\right]$	3 ± 3
cds [Å]	25 ± 6
$x_{\mathrm{Ti},1}$ $[a]$	0.100 ± 0.005
$x_{\mathrm{Ti},2}$ $[a]$	0.197 ± 0.004
$x_{\mathrm{O},1}$ $[a]$	0.06 ± 0.02
$x_{\mathrm{O},2}$ $[a]$	0.138 ± 0.012
$x_{\mathrm{O},3}$ $[a]$	0.131 ± 0.013
$x_{\mathrm{O},4}$ $[a]$	0.241 ± 0.014
$z_{\mathrm{Ti},1}$ $[c]$	0.706 ± 0.009
$z_{\mathrm{Ti},2}$ $[c]$	0.289 ± 0.006
$z_{\mathrm{O},1}$ $[c]$	0.37 ± 0.02
$z_{\mathrm{O},2}$ $[c]$	0.01 ± 0.03
$z_{\mathrm{O},3}$ $[c]$	0.71 ± 0.02
$z_{\mathrm{O},4}$ $[c]$	0.36 ± 0.03
Weight frac.	0.85
R_{w}	0.17

TiO_2-anatase

Table C5. Results for the anatase phase from two-phase refinement of *ex situ* PDF data for the pristine material of batch two. Please see Table C4 for results of the bronze phase.

**Variable [unit]**	**Value ± esd**
Scale factor	0.06 ± 0.05
a [Å]	3.8 ± 0.5
c [Å]	9.6 ± 0.2
$u_{\mathrm{iso,Ti}}$ $\left[\text{Å}^2\right]$	0.01 ± 0.01
$u_{\mathrm{iso,O}}$ $\left[\text{Å}^2\right]$	0.04 ± 0.08
δ_2 $\left[\text{Å}^{-2}\right]$	4 ± 2
cds [Å]	40 ± 40
Weight frac.	0.15
R_{w}	0.17

Batch two: cathode composite (two TiO_2 phases and a modified graphite phase)

TiO_2-bronze

Table C6. Results for the bronze phase from the refinement of the *ex situ* PDF data for the cathode composite. Please see Table C7 and Table C8 for the results of the anatase and modified graphite phases, respectively.

**Variable [unit]**	**Value ± esd**
Scale	0.24 ± 0.07
a [Å]	12.14 ± 0.09
b [Å]	3.75 ± 0.03
c [Å]	6.50 ± 0.07
β [°]	107.0 ± 0.8
$u_{\mathrm{iso,Ti}}$ $\left[\mathrm{Å}^2\right]$	0.008 ± 0.005
$u_{\mathrm{iso,O}}$ $\left[\mathrm{Å}^2\right]$	0.002 ± 0.005
δ_2 $\left[\mathrm{Å}^{-2}\right]$	1 ± 5
cds $\left[\mathrm{Å}\right]$	26 ± 8
$x_{\mathrm{Ti,1}}$ $[a]$	0.100 ± 0.006
$x_{\mathrm{Ti,2}}$ $[a]$	0.192 ± 0.006
$x_{\mathrm{O,1}}$ $[a]$	0.058 ± 0.010
$x_{\mathrm{O,2}}$ $[a]$	0.116 ± 0.011
$x_{\mathrm{O,3}}$ $[a]$	0.125 ± 0.010
$x_{\mathrm{O,4}}$ $[a]$	0.235 ± 0.010
$z_{\mathrm{Ti,1}}$ $[c]$	0.692 ± 0.014
$z_{\mathrm{Ti,2}}$ $[c]$	0.282 ± 0.010
$z_{\mathrm{O,1}}$ $[c]$	0.39 ± 0.02
$z_{\mathrm{O,2}}$ $[c]$	-0.3 ± 0.02
$z_{\mathrm{O,3}}$ $[c]$	0.71 ± 0.02
$z_{\mathrm{O,4}}$ $[c]$	0.35 ± 0.02
Weight frac.	0.80
R_{w}	0.25

TiO_2-anatase

Table C7. Results for the anatase phase from the refinement of the *ex situ* PDF data for the cathode composite. Please see Table C6 and Table C8 for the results of the bronze and modified graphite phases, respectively.

**Variable [unit]**	**Value ± esd**
Scale factor	0.06 ± 0.05
a [Å]	3.8 ± 0.5
c [Å]	9.6 ± 0.2
$u_{\mathrm{iso,Ti}}$ $\left[\text{Å}^2\right]$	0.01 ± 0.02
$u_{\mathrm{iso,O}}$ $\left[\text{Å}^2\right]$	0.2 ± 0.2
δ_2 $\left[\text{Å}^{-2}\right]$	4 ± 1
cds $\left[\text{Å}\right]$	40 ± 60
Weight frac.	0.15
R_{w}	0.17

Modified graphite

Table C8. Results for the modified graphite phase from the refinement of the *ex situ* PDF data for the cathode composite. Please see Table C6 and Table C7 for results of the bronze and anatase phases, respectively.

**Variable [unit]**	**Value ± esd**
Scale factor	0.04 ± 0.02
a [Å]	2.457 ± 0.013
$u_{\mathrm{iso,C}}$ $\left[\text{Å}^2\right]$	0.001 ± 0.003
δ_2 $\left[\text{Å}^{-2}\right]$	2.01 ± 0.03
cds $\left[\text{Å}\right]$	40 ± 40
R_{w}	0.17

3 nm: *operando* first frame (two TiO_2 phases and a modified graphite phase)

TiO_2-bronze

Table C9. Results for the bronze phase from the refinement of the first *operando* frame for the batch two material. Please see Table C10 and Table C11 for the results of the anatase and modified graphite phases, respectively.

**Variable [unit]**	**Value ± esd**
Scale	0.17 ± 0.06
a [Å]	12.16 ± 0.14
b [Å]	3.78 ± 0.04
c [Å]	6.54 ± 0.08
β [°]	107.3 ± 1.2
$u_{\mathrm{iso,Ti}}$ $\left[\text{Å}^2\right]$	0.006 ± 0.005
$u_{\mathrm{iso,O}}$ $\left[\text{Å}^2\right]$	0.01 ± 0.02
δ_2 $\left[\text{Å}^{-2}\right]$	1 ± 5
cds [Å]	26 ± 12
Weight frac.	0.80
R_{w}	0.35

TiO_2-anatase

Table C10. Results for the anatase phase from the refinement of the first *operando* frame for the batch two material. Please see Table C9 and Table C11 for the results of the bronze and modified graphite phases, respectively.

**Variable [unit]**	**Value ± esd**
Scale factor	0.06 ± 0.05
a [Å]	3.8 ± 0.5
c [Å]	9.6 ± 0.2
$u_{\mathrm{iso,Ti}}$ $\left[\text{Å}^2\right]$	0.01 ± 0.02
$u_{\mathrm{iso,O}}$ $\left[\text{Å}^2\right]$	0.2 ± 0.2
δ_2 $\left[\text{Å}^{-2}\right]$	4 ± 1
cds [Å]	40 ± 60
Weight frac.	0.15
R_{w}	0.17

Modified graphite

Table C11. Results for the modified graphite phase from the refinement of the first *operando* frame for the batch two material. Please see Table C9 and Table C10 for the results of the bronze and anatase phases, respectively.

**Variable [unit]**	**Value ± esd**
Scale factor	0.04 ± 0.02
a [Å]	2.457 ± 0.013
$u_{\mathrm{iso,C}}$ $\left[\text{Å}^2\right]$	0.001 ± 0.003
δ_2 $\left[\text{Å}^{-2}\right]$	2.01 ± 0.03
cds [Å]	40 ± 40
R_{w}	0.17
