# Supplementary material for: Operando pair distribution function analysis of nanocrystalline functional materials: the case of TiO2-bronze nanocrystals in Li-ion battery electrodes
Source: J Appl Crystallogr. 2024 Jul 29;57(Pt 4):1171–83. doi: 10.1107/S1600576724005624 (PMC11299615; doi:10.1107/S1600576724005624)
Supplement: Supplementary file 4 [file j-57-01171-sup4.pdf]

JOURNAL OF APPLIED CRYSTALLOGRAPHY

Volume 57 (2024)

Supporting information for article:

Operando pair distribution function analysis of nanocrystalline functional materials: the case of TiO2-bronze nanocrystals in Li-ion battery electrodes

Martin A. Karlsen, Jonas Billet, Songsheng Tao, Isabel Van Driessche, Simon J. L. Billinge and Dorthe B. Ravnsbæk

Appendix D
Noise-filtering using principal component analysis (PCA)

Real space

Explained variance ratio for PCA of $G(r)$

Fig. D displays the explained variance ratio and its cumulated version for PCA of the *operando* PDF data. A kink at four components is observable, indicating that four components are probably needed to describe the trends in the *operando* PDF data. The dashed line to the right indicates that four components are included if including 0.96 of the cumulated explained variance ratio for the PCA.

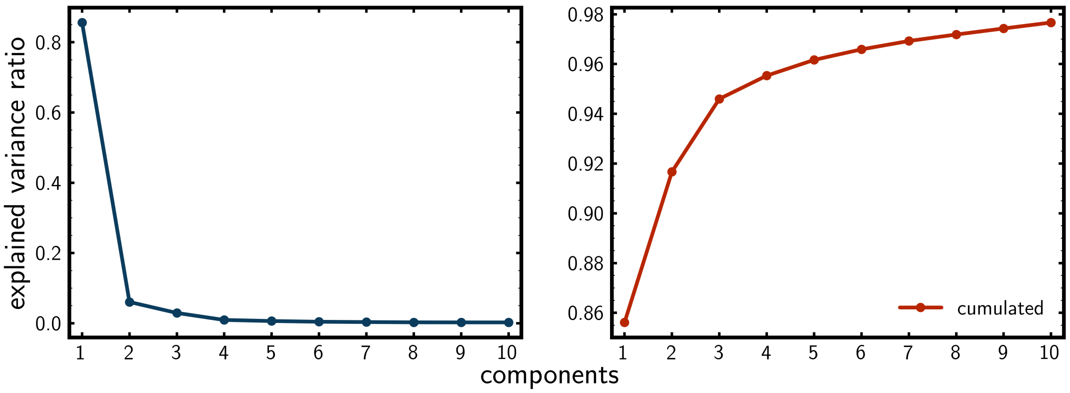

Fig. D1. Explained variance ratio and cumulated explained variance ratio as a function of the number of principal components. The value of 0.96 used for noise filtering is marked with a black dashed line.

Difference between experimental and PCA-reconstructed $G(r)$

Fig. D2 displays the between the experimental *operando* PDF data and the PCA-reconstructed data. As the difference appears structureless and relatively constant with r, it does look like noise, as desired.

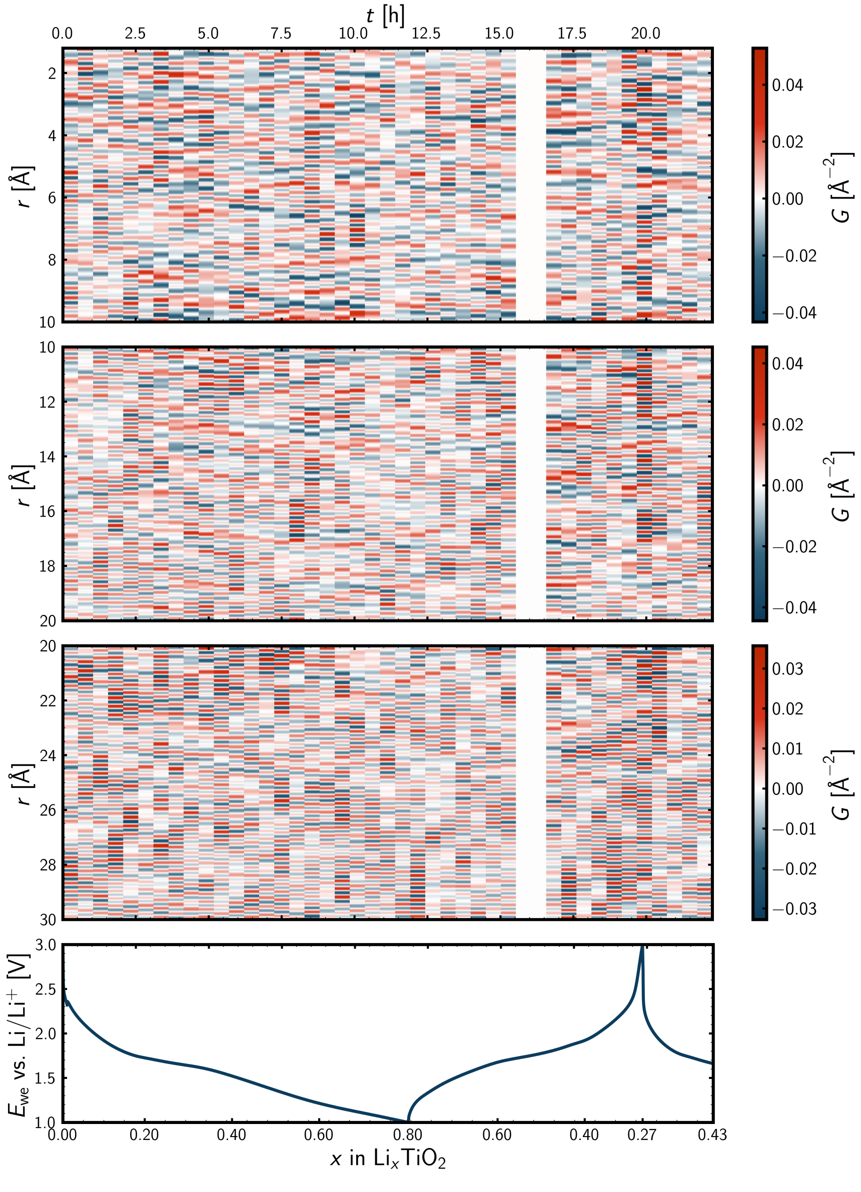

Fig. D2. Difference between the experimental and the PCA reconstruction of the reduced atomic pair distribution function, $G(r)$, together with the galvanostatic cycling. The $G(r)$ data have been divided into three r-regions, each region having its own colorbar to improve the visualization. The white column is due to absence of synchrotron x-rays during the *operando* experiment.

Pearson correlation coefficients between experimental and PCA-reconstructed $G(r)$

Fig. D3 displays the Pearson correlation coefficient between the experimental PDF and the PCA-reconstruction as a function of *operando* PDF frame number. All correlation coefficients are close to unity, revealing highly similarity as desired.

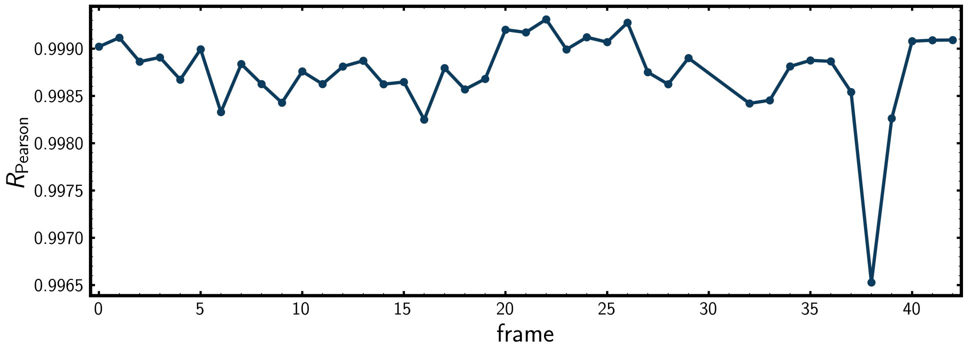

Fig. D3. Pearson correlation analysis of experimental and PCA-reconstructed reduced atomic pair distribution function data for all frames during the *operando* experiment.

Experimental, PCA-reconstructed, and difference $G(r)$

Fig. D4 displays the experimental PDF, the PCA-reconstruction, and their difference for the first *operando* PDF frame.

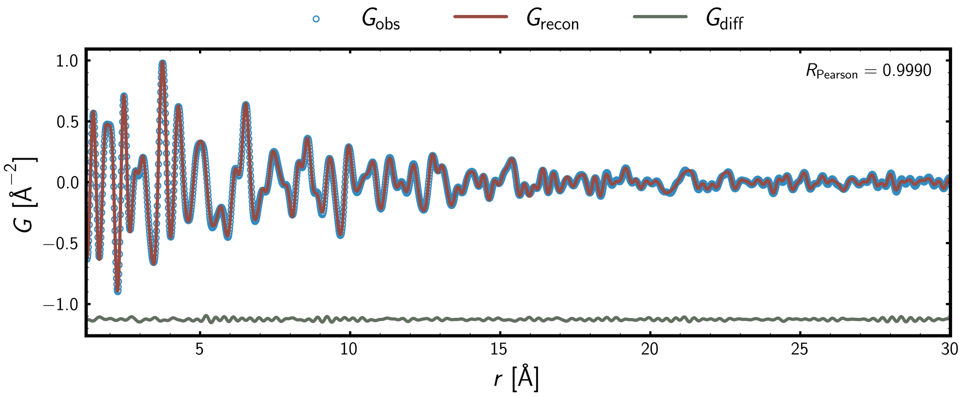

Fig. D4. Experimental reduced atomic pair distribution function data, PCA-reconstructed data, and the difference of the two, for the first frame of the *operando* experiment. The Pearson correlation coefficient is shown in the upper right corner.

Reciprocal space

Explained variance ratio for PCA of $F(Q)$

Fig. D shows the explained variance ratio and the cumulated explained variance ratio as a function of the number of principal components for the *operando* reduced total scattering structure function data. In both cases, a kink at four components is observed. For the cumulated explained variance ratio, a value of 0.93 for the PCA will ensure that enough of the trends in the experimental data are included in the reconstruction.

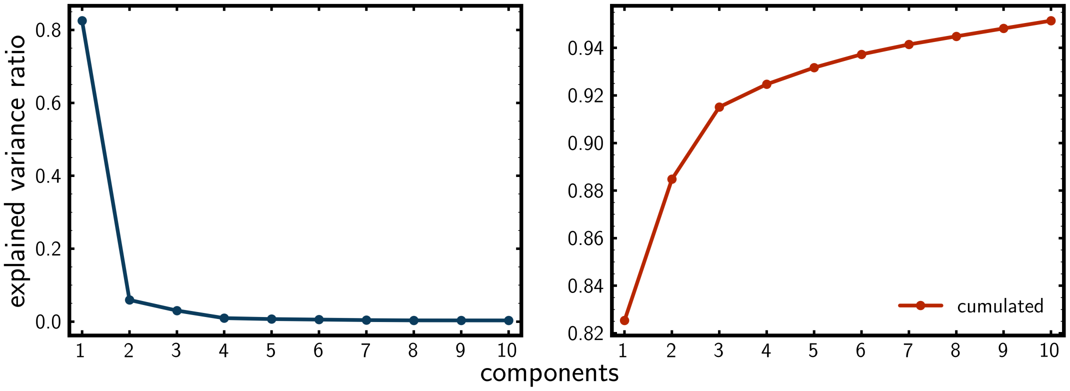

Fig. D5. Explained variance ratio and cumulated explained variance ratio as a function of the number of principal components. The value of 0.93 used for noise filtering is marked with a black dashed line.

Experimental and PCA-reconstructed $F(Q)$

Fig. D6 shows the PCA reconstruction of the $F(Q)$ data together with the galvanostatic cycling data. That the PCA serves as a noise filter is clearly seen for the high-Q region, as the unfiltered signal suffers from a lower signal-to-noise ratio there, resulting from the x-ray atomic form factor.

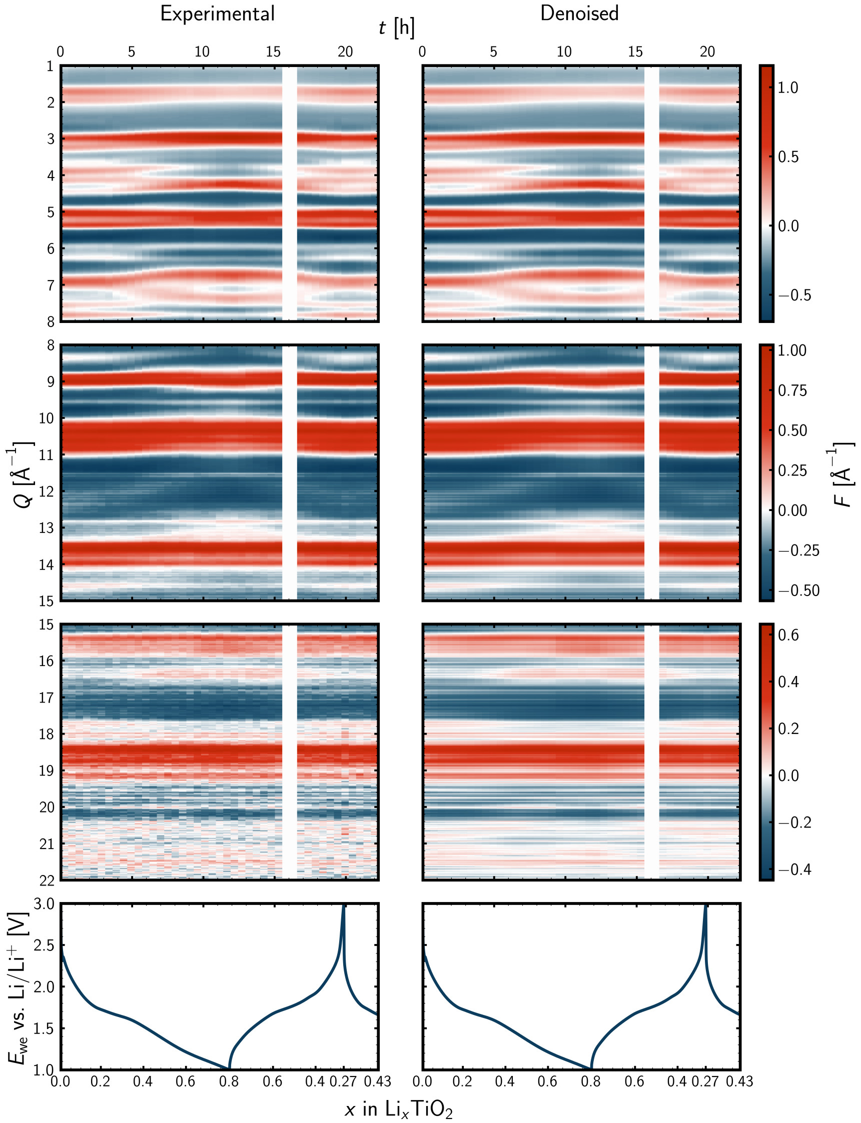

Fig. D6. Experimental (left) and noise-filtered (PCA-reconstructed, right) *operando* reduced total scattering structure function, $F(Q)$, together with the galvanostatic cycling. The $F(Q)$ data have been divided into three Q-regions, each region having its own colorbar to improve the visualization. The white column is due to absence of synchrotron x-rays during the *operando* experiment.

Difference between experimental and PCA-reconstructed $F(Q)$

Fig. D7 shows the difference between the experimental data and the PCA reconstruction, i.e., the filtered noise, of the reduced total scattering structure function, $F(Q)$, together with the galvanostatic cycling data. As desired, the part of the signal that is filtered appears structureless, i.e, behaves as noise. From the relative trends within each subplot, the noise-level increases momentum transfer, Q. Comparing the color scales of the subplots, the noise-level also increases with momentum transfer in absolute terms. This arises from the multiplication with Q during the last step in the data processing to obtain the reduced total scattering structure function, $I(Q) \rightarrow S(Q) \rightarrow F(Q) = Q[S(Q) - 1]$.

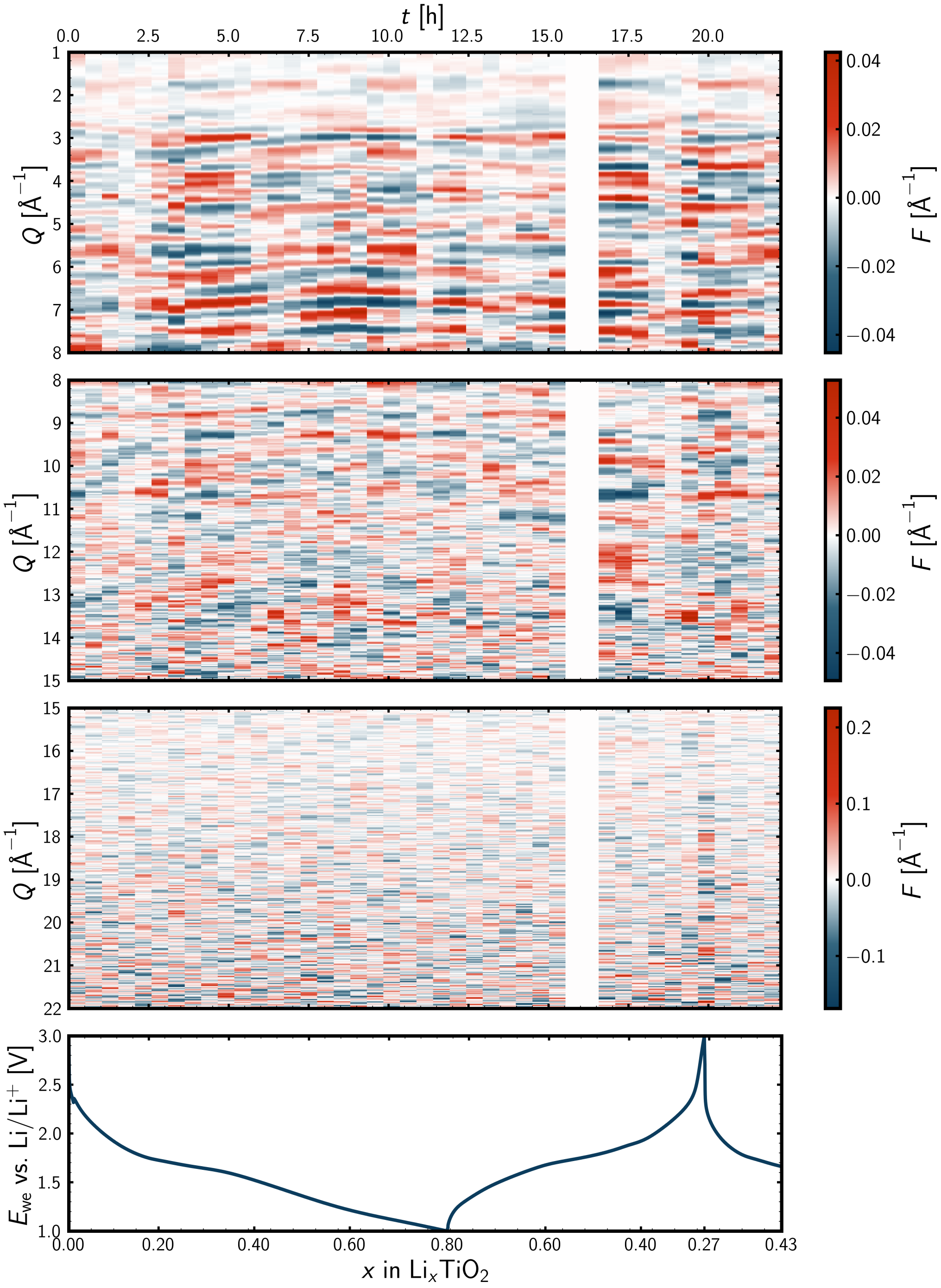

Fig. D7. Difference between the experimental and the PCA reconstruction of the reduced total scattering structure function, $F(Q)$, together with the galvanostatic cycling. The $F(Q)$ data have been divided into three Q-regions, each region having its own colorbar to improve the visualization. The white column is due to absence of synchrotron x-rays during the *operando* experiment.

Pearson correlation coefficients between experimental and PCA-reconstructed $F(Q)$

Fig. D8 displays the Pearson correlation coefficient for the experimental and PCA-reconstructed reduced total scattering structure function data as a function of frame number during the *operando* experiment. All correlation coefficients are above 0.99 and the patterns are practically speaking identical. The minor deviation from a value of unity reflects that the small difference from the filtering of the experimental data.

Frame 38 during the last part of the experiment stands out. However, re-inspection of the metadata of the raw data revealed that the exposure time for this frame was only one-fourth of that of the remaining frames, i.e., only one minute instead of four minutes.

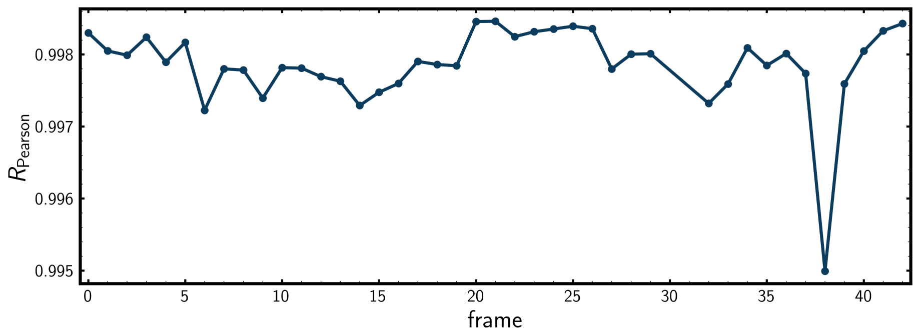

Fig. D8. Pearson correlation analysis of experimental and PCA-reconstructed reduced total scattering structure function data for all frames during the *operando* experiment.

Experimental data, PCA reconstruction, and difference Fig. D9 shows a plot of the experimental, the PCA-reconstructed data, and the difference of the two, for the reduced total scattering structure function for the first frame of the *operando* experiment. The Pearson correlation coefficient is also displayed. From the difference curve, it is evident that more noise is filtered from the experimental data at high values of momentum transfer, Q.

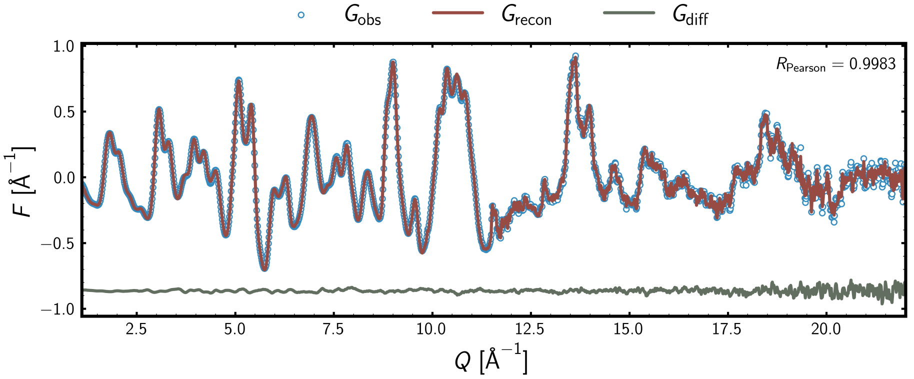

Fig. D9. Experimental reduced total scattering structure function data, PCA-reconstructed data, and the difference of the two, for the first frame of the *operando* experiment. The Pearson correlation coefficient is shown in the upper right corner.
