# Supplementary material for: Operando pair distribution function analysis of nanocrystalline functional materials: the case of TiO2-bronze nanocrystals in Li-ion battery electrodes
Source: J Appl Crystallogr. 2024 Jul 29;57(Pt 4):1171–83. doi: 10.1107/S1600576724005624 (PMC11299615; doi:10.1107/S1600576724005624)
Supplement: Supplementary file 5 [file j-57-01171-sup5.pdf]

JOURNAL OF
APPLIED
CRYSTALLOGRAPHY

Volume 57 (2024)

Supporting information for article:

Operando pair distribution function analysis of nanocrystalline functional materials: the case of TiO2-bronze nanocrystals in Li-ion battery electrodes

Martin A. Karlsen, Jonas Billet, Songsheng Tao, Isabel Van Driessche, Simon J. L. Billinge and Dorthe B. Ravnsbæk

Appendix E
similarityMapping: Pearson correlation analysis for *operando* PDF data

From the r-dependent Pearson correlation analyses in Figs. E1-E3, similar trends are observed as for the full range in Fig. 6. Comparing the values of all the color scales reveals that higher dissimilarity (lower correlation coefficients) for the intermediate r-range from 10 Å to 20 Å in Fig. E2. In the low-r range in Fig. E1, the PDFs are highly similar, as the phases are made from the same building blocks, i.e., TiO_6-octahedra. In the high-r range in Fig. E3, the signal-noise-ratio is low that the correlation analysis is less sensitive to structural differences.

From 0 to 10 Å

Fig. E1 shows the result of Pearson correlation analysis for the r range from 0 to 10 Å. The result is highly similar to Fig. 6, only with a little less contrast, i.e., the minimum R-value observed is a little higher in Fig. E1. This reflects the high similarity of the PDFs, and therefore the atomic structures, in this r-region.

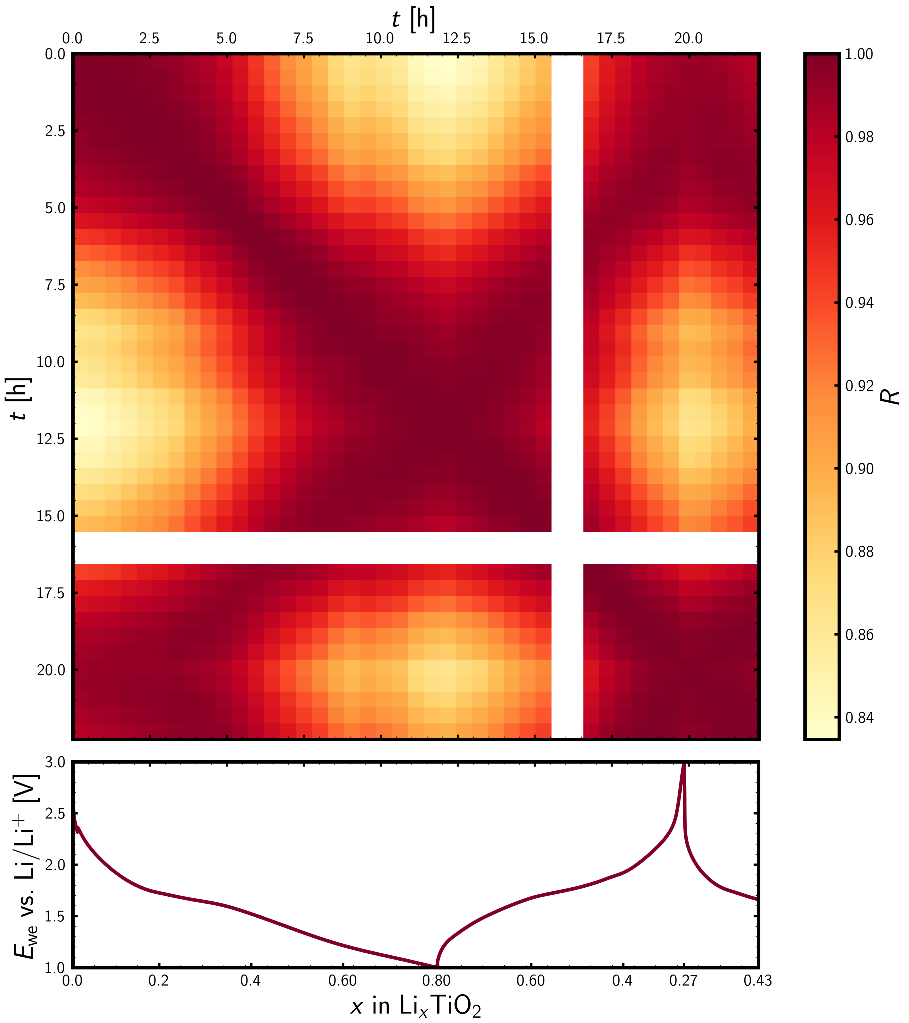

Fig. E1. Top: Pearson cross-correlation matrix for the reduced atomic pair distribution functions, $G(r)$, of the *operando* experiment, with the corresponding time, t, on the axes. The correlation analysis was conducted for the r-range from 0 to 10 Å. The value of the Pearson correlation coefficient, R, is given by the colorbar to the right. Bottom: voltage profile with the electrochemical potential of the working electrode, E_{we} Li/Li$^+$, as a function of state of charge, x, in Li$_x$TiO$_2$.

From 10 to 20 Å

Fig. E2 shows the result of Pearson correlation analysis for the r range from 10 to 20 Å. Even though the appearance is highly similar to Fig. 6, a significantly higher contrast is observed, i.e., the minimum R-value observed is significantly lower in Fig. E2. This reflects the higher dissimilarity of the PDFs, and therefore the atomic structures, for this r-range. This should be exptected, e.g., when comparing to the very local range from 0 to 10 Å in Fig. E1.

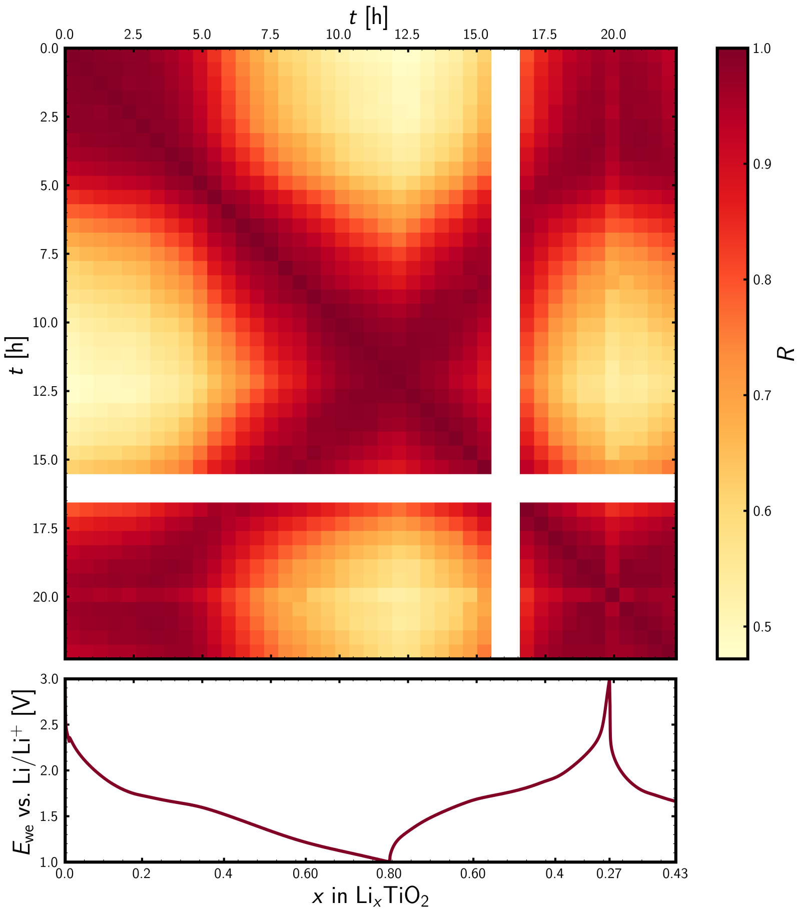

Fig. E2. Top: Pearson cross-correlation matrix for the reduced atomic pair distribution functions, $G(r)$, of the *operando* experiment, with the corresponding time, t, on the axes. The correlation analysis was conducted for the r-range from 10 to 20 Å. The value of the Pearson correlation coefficient, R, is given by the colorbar to the right. Bottom: voltage profile with the electrochemical potential of the working electrode, E_{we} Li/Li$^+$, as a function of state of charge, x, in Li$_x$TiO$_2$.

From 20 to 30 Å

Fig. E3 shows the result of Pearson correlation analysis for the r range from 20 to 30 Å. Even though the appearance is sort of similar to Fig. 6, the level of noise is much higher. This reflects the lower signal-to-noise ratio for this r-range due to the dampening of the PDFs, together with the increased overlap of correlation peaks with increasing r.

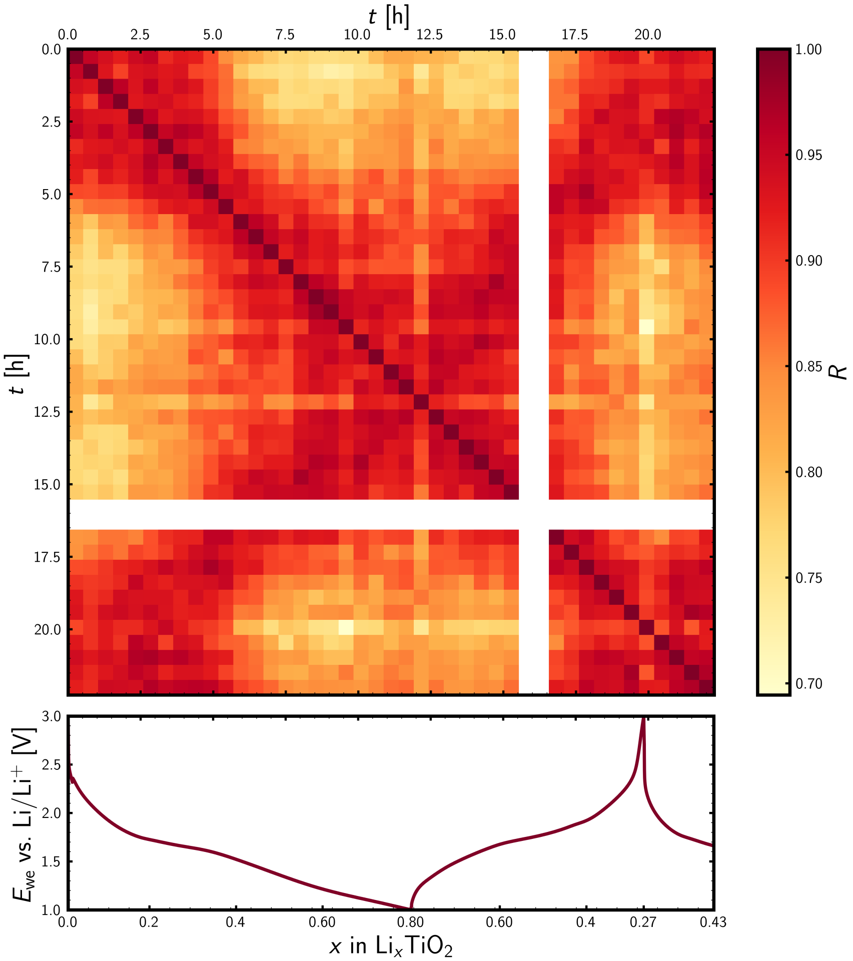

Fig. E3. Top: Pearson cross-correlation matrix for the reduced atomic pair distribution functions, $G(r)$, of the *operando* experiment, with the corresponding time, t, on the axes. The correlation analysis was conducted for the r-range from 20 to 30 Å. The value of the Pearson correlation coefficient, R, is given by the colorbar to the right. Bottom: voltage profile with the electrochemical potential of the working electrode, E_{we} Li/Li$^+$, as a function of state of charge, x, in Li_xTiO_2.
