# Supplementary material for: Operando pair distribution function analysis of nanocrystalline functional materials: the case of TiO2-bronze nanocrystals in Li-ion battery electrodes
Source: J Appl Crystallogr. 2024 Jul 29;57(Pt 4):1171–83. doi: 10.1107/S1600576724005624 (PMC11299615; doi:10.1107/S1600576724005624)
Supplement: Supplementary file 6 [file j-57-01171-sup6.pdf]

JOURNAL OF APPLIED CRYSTALLOGRAPHY

Volume 57 (2024)

Supporting information for article:

Operando pair distribution function analysis of nanocrystalline functional materials: the case of TiO2-bronze nanocrystals in Li-ion battery electrodes

Martin A. Karlsen, Jonas Billet, Songsheng Tao, Isabel Van Driessche, Simon J. L. Billinge and Dorthe B. Ravnsbæk

Appendix F
nmfMapping: Non-negative matrix factorization for *operando* data

From the various NMF analyses of reciprocal and real space data in Fig. 7 and Figs. F1-F8, a physical interpretation of the beahvior of the NMF weights is possible up to four components. When using five components, the behavior of the NMF weights cannot be accounted for in a physically meaningful way. Conducting NMF analysis in reciprocal and real space results in similar behavior of the NMF weights, though the relative size of the weights differ a little. In reciprocal space, the change in the weights is observed to be larger. The interpretation of this is that the phases differ more in reciprocal space than in real space, as should be expected. However, due to the nanosize of the materials, extraction of structural information through modelling must be done in real space through PDF analysis.

Two components: real space

Fig. F1 displays the output from the nmfMapping app using two components for the reduced atomic pair distribution function data, together with the Galvanostatic cycling. From the behavior of the weights and the electrochemistry, it can be seen that the two components represent at Li-poor (1, navy) and Li-rich (2, red) states, respectively. From the extent of the components, it can be seen that the red components terminates a little earlier, indicating a shorter length of structural coherence for the Li-rich state. Since only one component is present for the pristine material, the number of components used is too low, as the *ex situ* analysis revealed two phases, TiO_2-bronze and TiO_2-anatase.

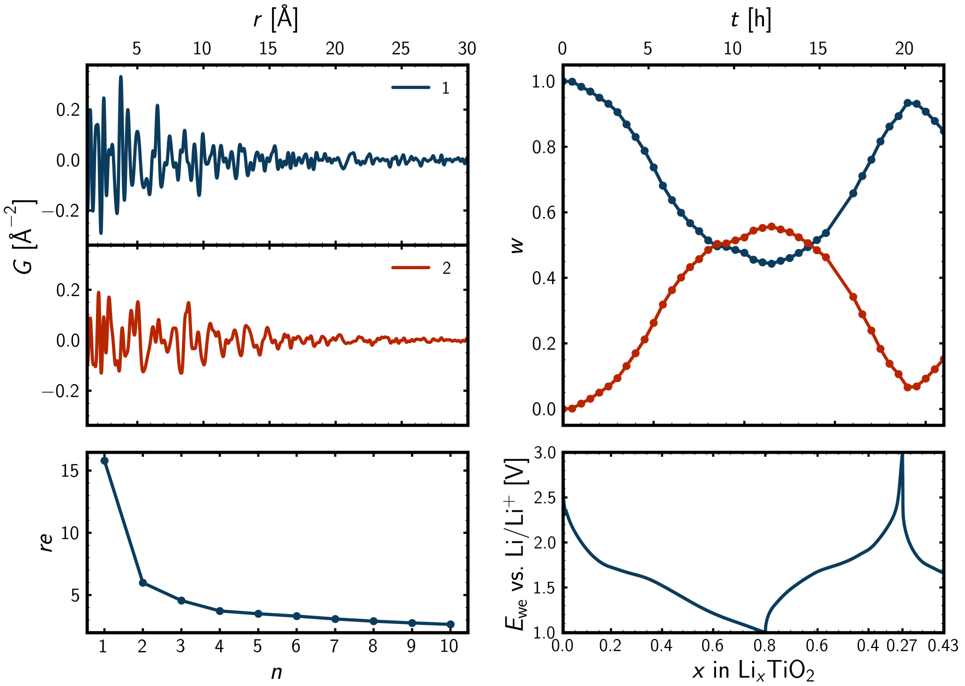

Fig. F1. Output from nmfMapping at PDFitc when setting the threshold for the number of components to two. Top left: for each component, the reduced atomic pair distribution function, $G(r)$, is shown. Bottom left: the reconstruction error, re, as a function of the number of components, n. Top right: NMF weights, w, as a function of time, t, in hours, h. Bottom right: voltage profile. The electrochemical potential of the working electrode, E_{we} vs. Li/Li^+, as a function of the state of charge, x in Li_xTiO_2, during the *operando* experiment.

Two components: reciprocal space

Fig. F2 displays the output from the nmfMapping app using two components for the reduced total scattering structure function data, together with the Galvanostatic cycling. As is the case for Fig. F1, the two components represents Li-poor (1, navy) and Li-rich (2, red) states, respectively. The behavior of the weights is comparable to that for the PDF data in Fig. F1, though the changes in weights are a little more pronounced for the $F(Q)$ data here, which might indicate that it is easier to distinguish the two components (phases) in reciprocal space.

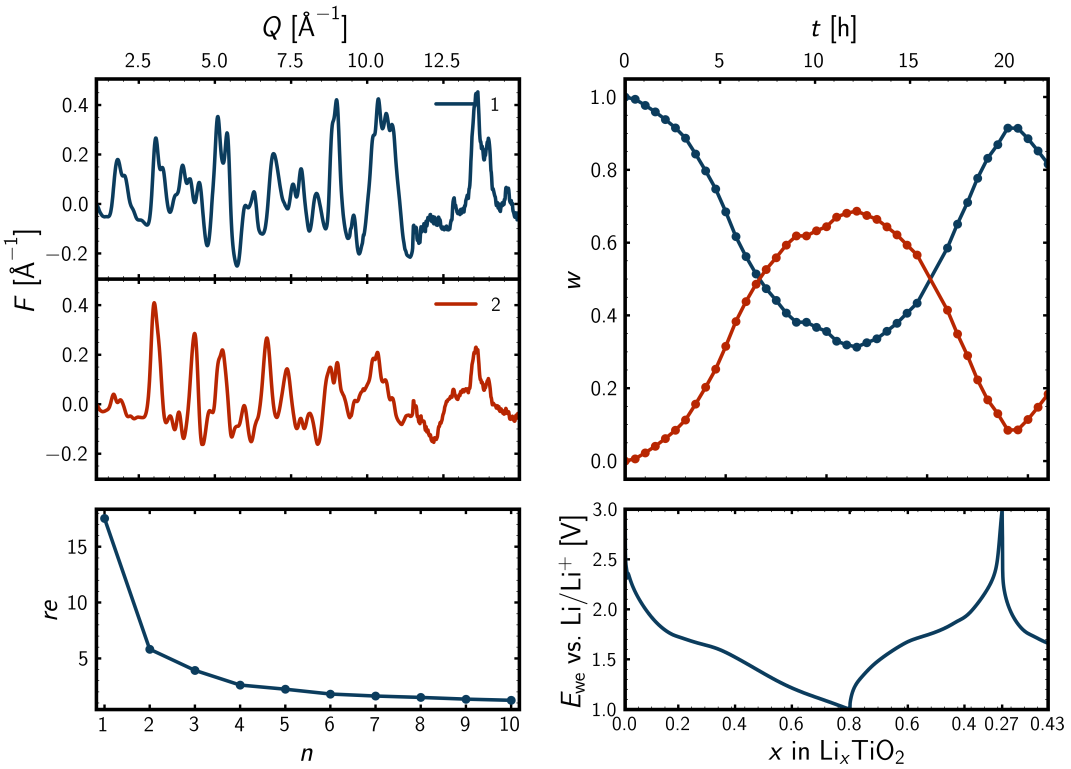

Fig. F2. Output from nmfMapping at PDFitc when setting the threshold for the number of components to two. Top left: for each component, the reduced total scattering structure function, $F(Q)$, is shown. Bottom left: the reconstruction error, re, as a function of the number of components, n. Top right: NMF weights, w, as a function of time, t, in hours, h. Bottom right: voltage profile. The electrochemical potential of the working electrode, E_{we} vs. Li/Li^{+}, as a function of the state of charge, x in Li_xTiO_2, during the *operando* experiment.

Three components: real space

Fig. F3 displays the output from the nmfMapping app using three components for the reduced atomic pair distribution function data, together with the Galvanostatic cycling. In addition to the Li-poor (1, navy) and Li-rich (2, red) components, a component of intermediate degree of lithiation (3, green) appears as an intermediate during both discharge and charge. It is worth to note that the component seems to be absent at complete charged and discharged states and that its evolution appears to be somewhat reversible.

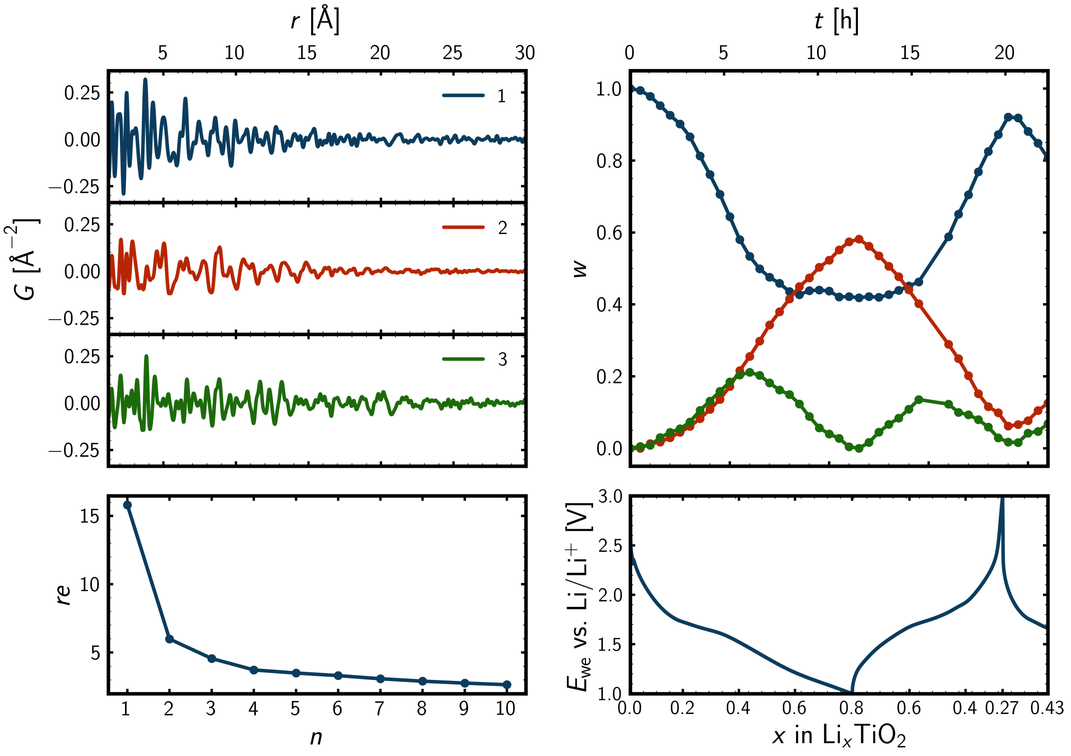

Fig. F3. Output from nmfMapping at PDFitc when setting the threshold for the number of components to three. Top left: for each component, the reduced atomic pair distribution function, $G(r)$, is shown. Bottom left: the reconstruction error, re, as a function of the number of components, n. Top right: NMF weights, w, as a function of time, t, in hours, h. Bottom right: voltage profile. The electrochemical potential of the working electrode, E_{we} vs. Li/Li^+, as a function of the state of charge, x in Li_xTiO_2, during the *operando* experiment.

Three components: reciprocal space

Fig. F4 displays the output from the nmfMapping app using three components for the reduced total scattering structure function data, together with the Galvanostatic cycling. The relative behavior of the weights is again comparable to that observe in real space in Fig. F3, however, as observed in Figs. F1 and F2, the changes in the weights are more pronounced for the $F(Q)$ data, indicating that it is easier to distinguish the three components (phases) in reciprocal space.

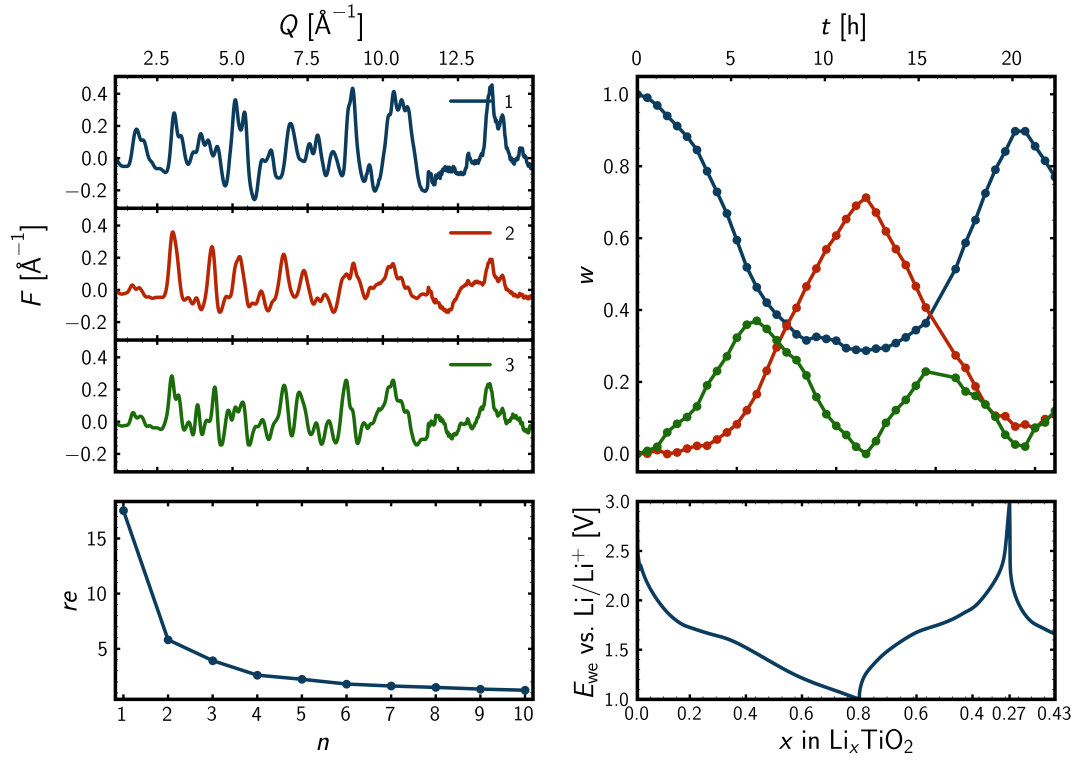

Fig. F4. Output from nmfMapping at PDFitc when setting the threshold for the number of components to three. Top left: for each component, the reduced total scattering structure function, $F(Q)$, is shown. Bottom left: the reconstruction error, re, as a function of the number of components, n. Top right: NMF weights, w, as a function of time, t, in hours, h. Bottom right: voltage profile. The electrochemical potential of the working electrode, E_{we} vs. Li/Li^{+}, as a function of the state of charge, x in Li_xTiO_2, during the *operando* experiment.

Four components: real space

Fig. F5 shows the output components when using four component for the NMF analysis. Each of the components are plotted together with the calculated PDF of the phase, which there are interpreted to represent. The .cif files used to calculate the PDFs are those obtained from the structureMining app at PDFitc for the *ex situ* PDF data in Appendix B. In Fig. F5, it is clearly seen that the NMF components do not resemble the calculated PDFs one-to-one. Reasons for this are the multicomponent nature of the *operando* data, where C-C correlations at $r = 1.4\,\text{Å}$ are seen for all four components. In addition, the NMF components are only allowed to reconstruct the experimental data through linear combinations using the NMF weights. Hence, the structural changes expected to occur for each of the individual phases are only allowed to be captured by varying the scale factor (NMF weight). This also explains why each individual NMF component might not resemble the corresponding PDF calculated from a .cif file representing a static structure. The lack of one-to-one correspondence between the NMF components and the PDFs of the individual phases also explains why structureMining does not work on the NMF components.

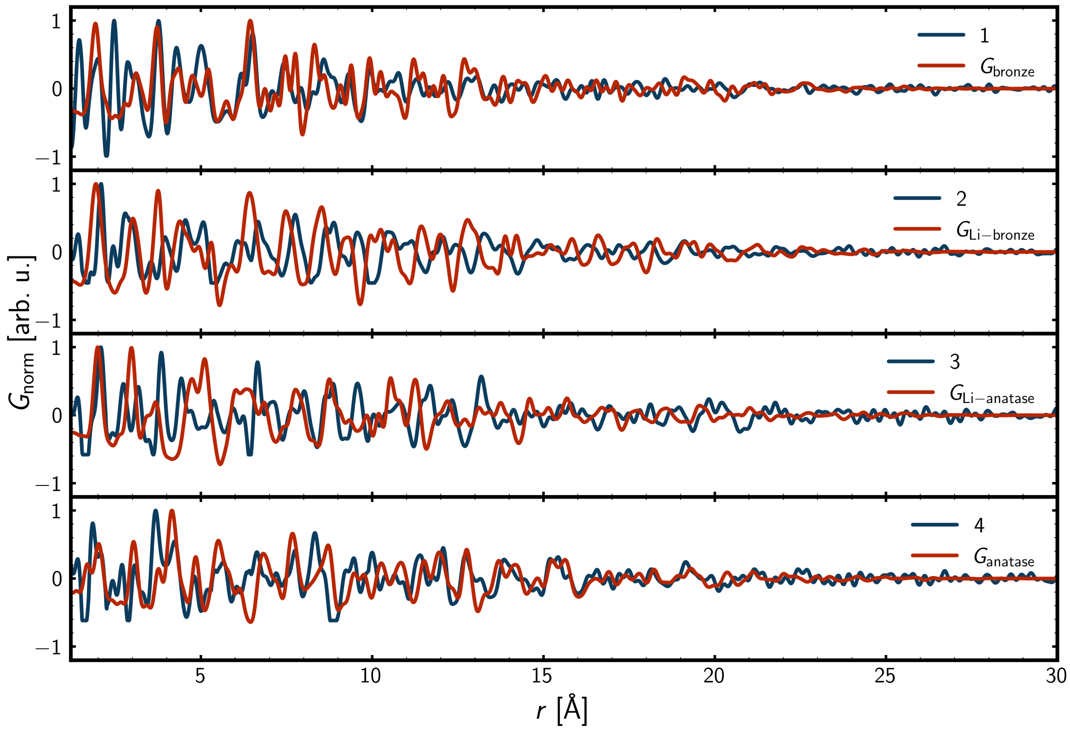

Fig. F5. Normalized NMF components and calculated PDFs of the phases, which the NMF components are interpreted to represent shown, G_{norm} [arb. u.], as a function of the interatomic distance, r, in Ångström, Å.

Four components: reciprocal space

Fig. F6 displays the output from the nmfMapping app using four components for the reduced total scattering structure function data, together with the Galvanostatic cycling. Comparing to Fig. 7, the relative beahvior of the weights are similar, though the changes in weights are more pronounced for the $F(Q)$ data here, pointing towards that it is easier to distinguish the four components (phases) in reciprocal space.

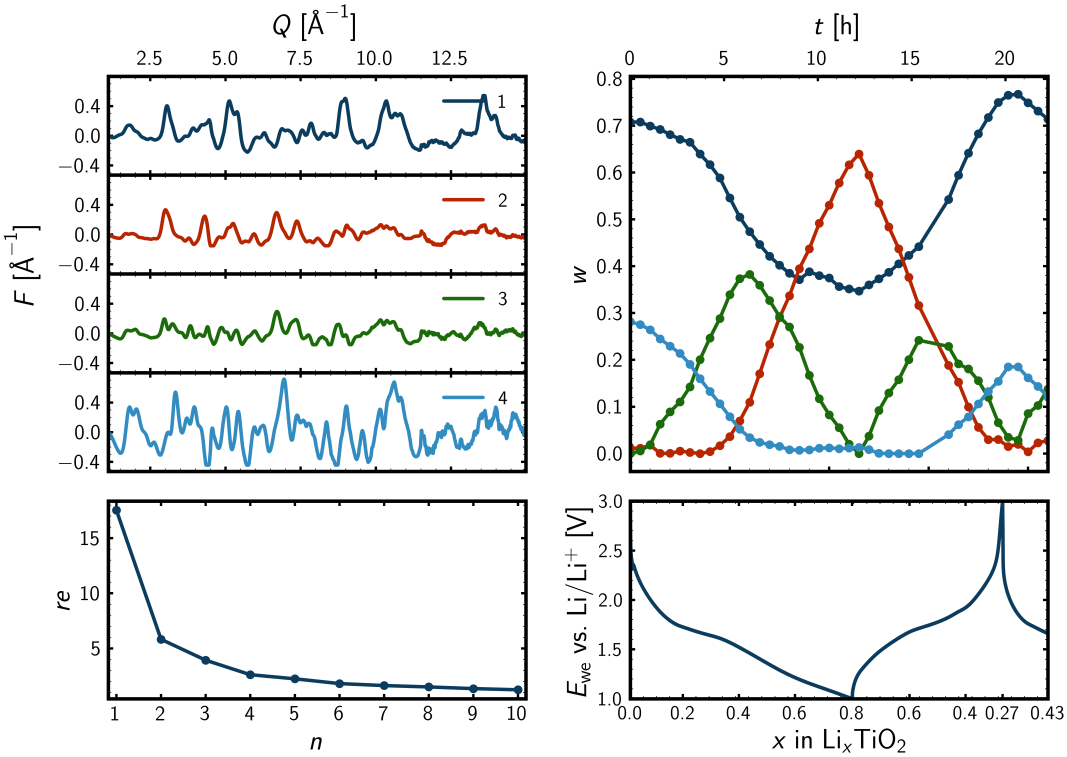

Fig. F6. Output from nmfMapping at PDFitc when setting the threshold for the number of components to four. Top left: for each component, the reduced total scattering structure function, $F(Q)$, is shown. Bottom left: the reconstruction error, re, as a function of the number of components, n. Top right: NMF weights, w, as a function of time, t, in hours, h. Bottom right: voltage profile. The electrochemical potential of the working electrode, E_{we} vs. Li/Li^{+}, as a function of the state of charge, x in Li_xTiO_2, during the *operando* experiment.

Five components: real space

Fig. F7 displays the output from the nmfMapping app using five components for the reduced atomic pair distribution function data, together with the Galvanostatic cycling. From the appearance of the components and the behavior of the weights, it is no longer possible to make sense of the output. Comparing to the weights of the analysis using four components in Fig. 7, it looks like the fifth component in grey accounts for some of the signal that the fourth component in light blue otherwise would do. Hence, it is not possible to interpret the NMF output in a meaningful way beyond four components, as indicated by the reconstruction error as a function of the number of components in Fig. F7 bottom left.

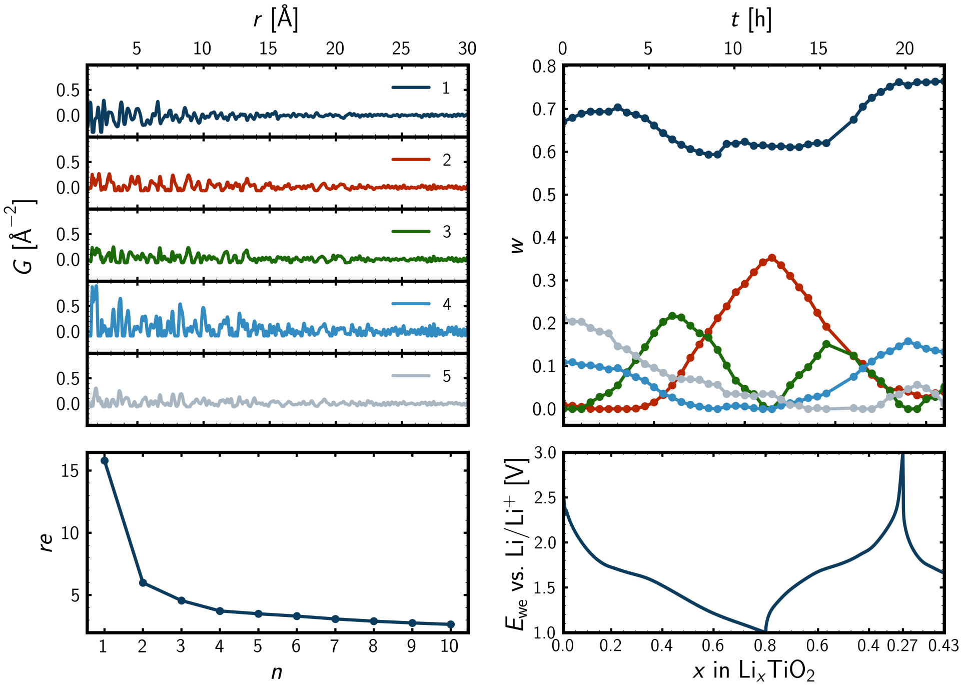

Fig. F7. Output from nmfMapping at PDFitc when setting the threshold for the number of components to five. Top left: for each component, the reduced atomic pair distribution function, $G(r)$, is shown. Bottom left: the reconstruction error, re, as a function of the number of components, n. Top right: NMF weights, w, as a function of time, t, in hours, h. Bottom right: voltage profile. The electrochemical potential of the working electrode, $E_{\rm we}$ vs. Li/Li^+, as a function of the state of charge, x in Li_xTiO_2, during the *operando* experiment.

Five components: reciprocal space

Fig. F8 displays the output from the nmfMapping app using five components for the reduced total scattering structure function data, together with the Galvanostatic cycling. As is the case in Fig. F7, it is no longer possible to make physical sense of the NMF output. The magnitude of the signal of the fifth component is much smaller than the first four components, indicating that the algorithm might start to include noise into the matrix decompomsition, which is undesirable. As concluded for the real space data in Fig. F7, it is not possible to interpret the NMF output in a meaningful way beyond four components, as indicated by the reconstruction error as a function of the number of components in Fig. F8 bottom left.

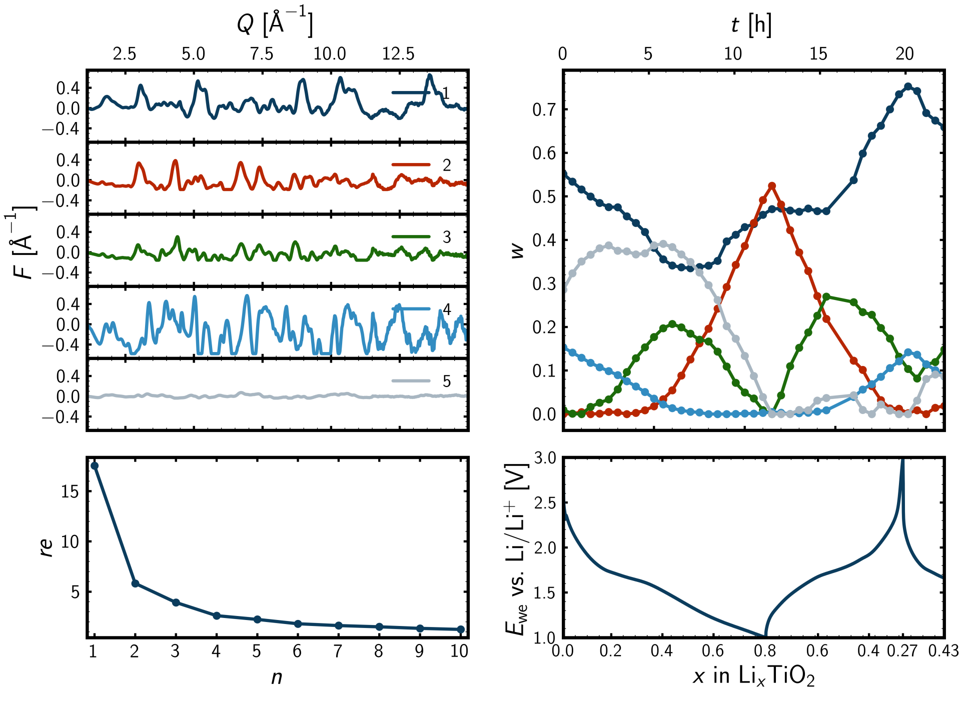

Fig. F8. Output from nmfMapping at PDFitc when setting the threshold for the number of components to five. Top left: for each component, the reduced total scattering structure function, $F(Q)$, is shown. Bottom left: the reconstruction error, re, as a function of the number of components, n. Top right: NMF weights, w, as a function of time, t, in hours, h. Bottom right: voltage profile. The electrochemical potential of the working electrode, E_{we} vs. Li/Li^{+}, as a function of the state of charge, x in Li_xTiO_2, during the *operando* experiment.
