# Supplementary material for: Operando pair distribution function analysis of nanocrystalline functional materials: the case of TiO2-bronze nanocrystals in Li-ion battery electrodes
Source: J Appl Crystallogr. 2024 Jul 29;57(Pt 4):1171–83. doi: 10.1107/S1600576724005624 (PMC11299615; doi:10.1107/S1600576724005624)
Supplement: Supplementary file 7 [file j-57-01171-sup7.pdf]

JOURNAL OF APPLIED CRYSTALLOGRAPHY

Volume 57 (2024)

Supporting information for article:

Operando pair distribution function analysis of nanocrystalline functional materials: the case of TiO2-bronze nanocrystals in Li-ion battery electrodes

Martin A. Karlsen, Jonas Billet, Songsheng Tao, Isabel Van Driessche, Simon J. L. Billinge and Dorthe B. Ravnsbæk

Appendix G
Operando PDF modelling

Pristine

Fig. G1 displays the PDF fit of the first *operando* frame. It can be seen that the modified graphite phase accounts for a significant part of the total signal, comparable to that of the bronze phase, whereas the minor anatase phase contributes less.

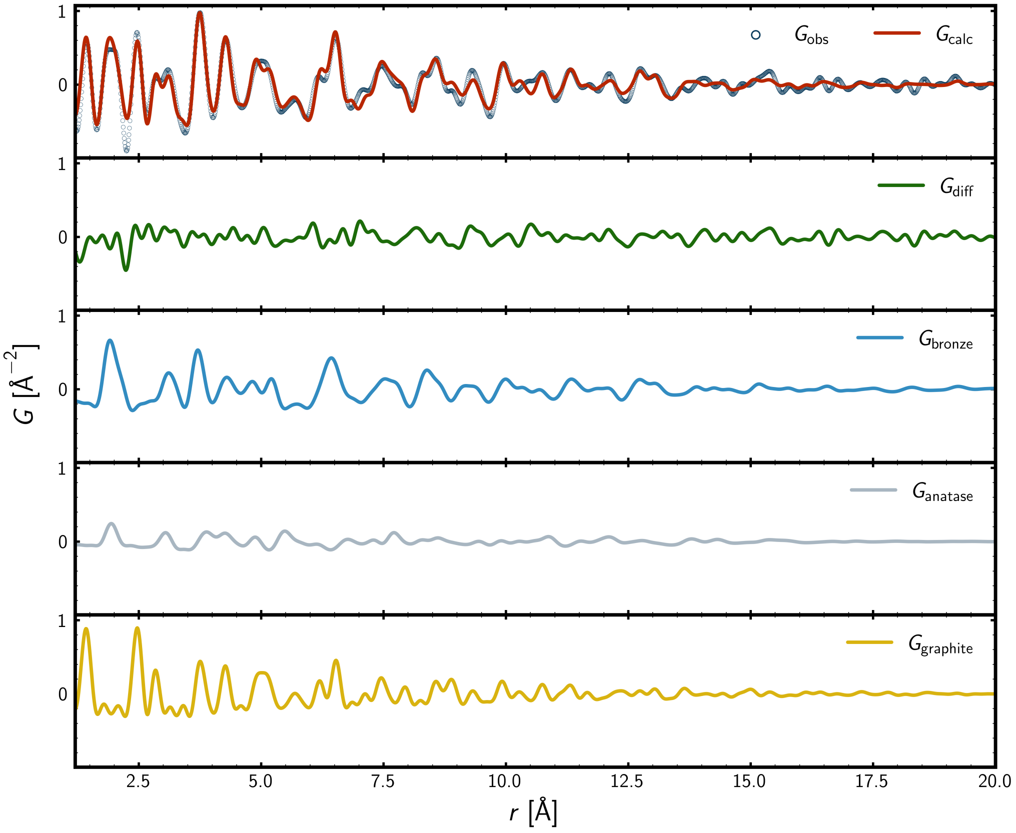

Fig. G1. PDF fit for first *operando* frame. The observed and calculated PDFs are shown topmost as blue circles and a red line, respectively. Below, the difference of the observed and calculated PDFs is shown in green. The calculated PDFs of the bronze, anatase, and modified graphite phases are shown in light blue, grey, and yellow, respectively.

Midpoint of initial discharge

Fig. G2 displays the state at the midpoint of the initial discharge. The more disordered nature of the minor TiO_2-anatase (grey) and lithiated anatase (yellow) phases are clearly evident from both the severe broadening and dampning of the PDFs.

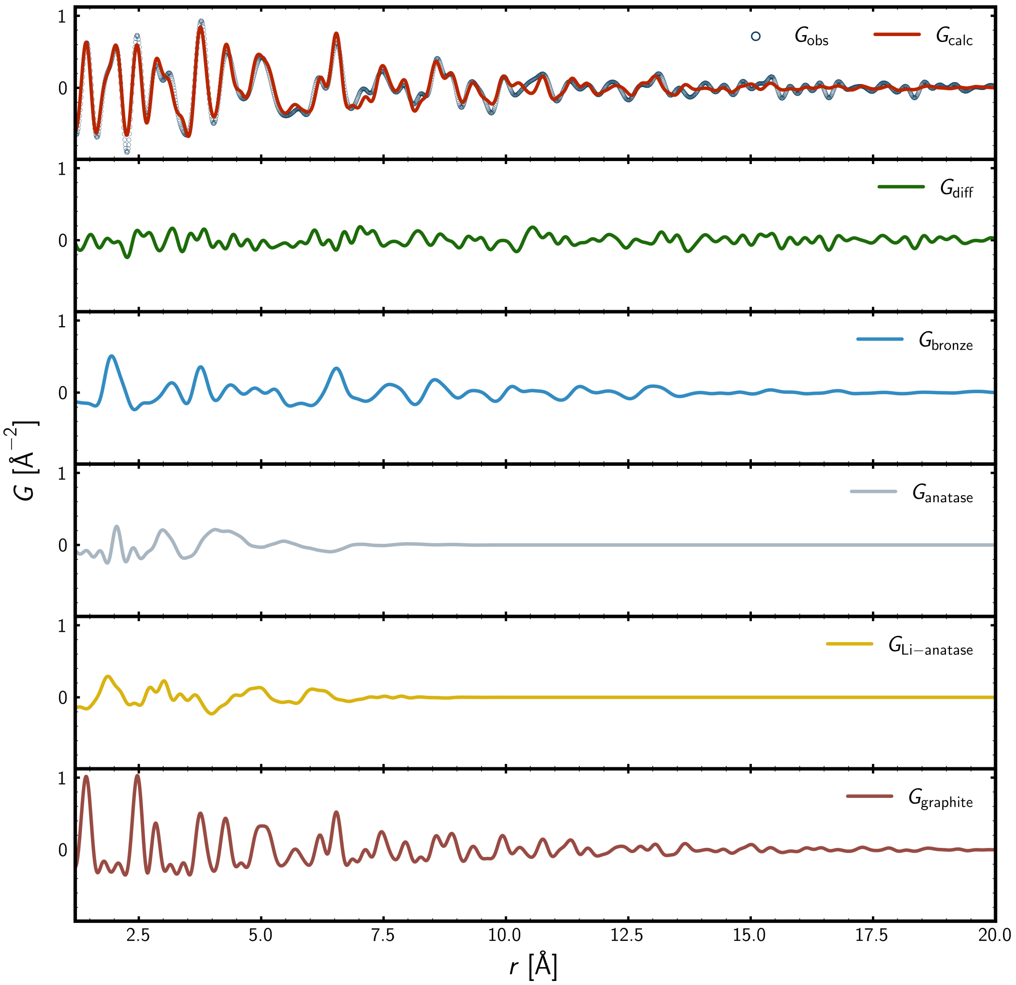

Fig. G2. PDF fit for *operando* frame at the midpoint of the initial discharge. The observed and calculated PDFs are shown topmost as blue circles and a red line, respectively. Below, the difference of the observed and calculated PDFs is shown in green. The calculated PDFs of the bronze, anatase, lithiated anatase, and modified graphite phases are shown in light blue, grey, yellow, and maroon, respectively.

End of initial discharge

Fig. G3 represents the state at the end of the initial discharge. From the difference curve in green, structural features not decribed by the model are clearly evident.

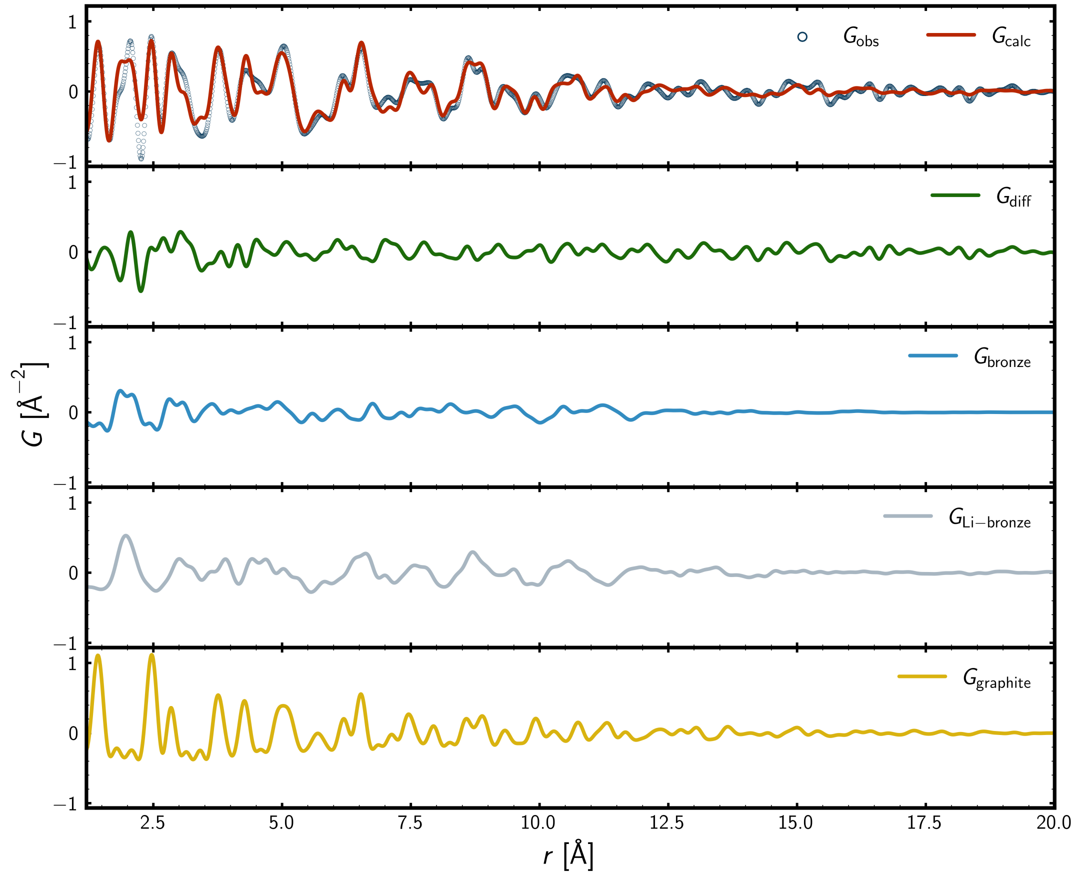

Fig. G3. PDF fit for *operando* frame at the midpoint of the initial discharge. The observed and calculated PDFs are shown topmost as blue circles and a red line, respectively. Below, the difference of the observed and calculated PDFs is shown in green. The calculated PDFs of the bronze, lithiated bronze, and modified graphite phases are shown in light blue, grey, and yellow, respectively.

End of charge

Fig. G4 represents the state at the end of the charge. Comparing to the fit for the pristine material in Fig. G1, the TiO_2-anatase phase in grey appears more disordered from the severe broadening and dampening of the PDF.

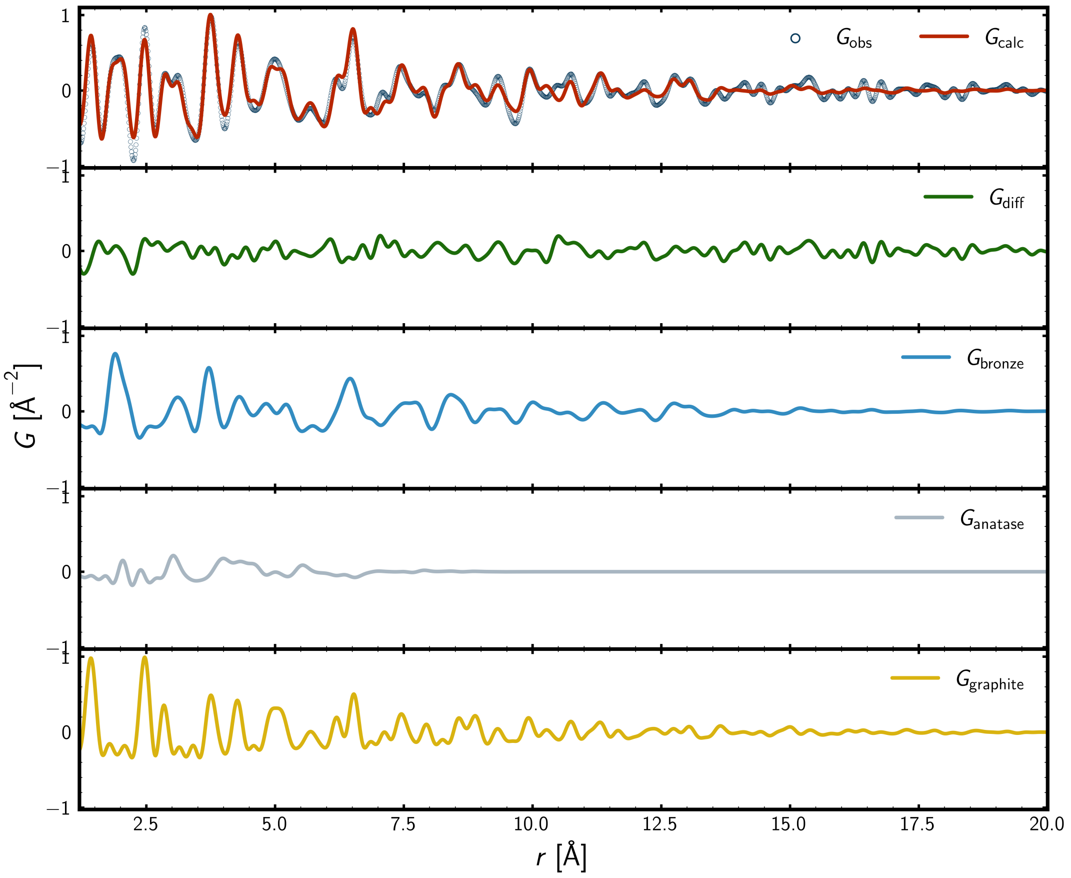

Fig. G4. PDF fit for *operando* frame at the end of the charge. The observed and calculated PDFs are shown topmost as blue circles and a red line, respectively. Below, the difference of the observed and calculated PDFs is shown in green. The calculated PDFs of the bronze, anatase, and modified graphite phases are shown in light blue, grey, and yellow, respectively.

TiO_2-bronze unit cell parameters

Fig. G5 displays the refined lattice parameters for the TiO_2-bronze phase. A discontinuity is observed for the a and c parameters during the initial discharge around $x \approx 0.4$, where the Li_xTiO_2-anatase phase is included in the refinement. Another discontinuity during the initial discharge is observed for all four lattice parameters around $x \approx 0.65$, where the Li_xTiO_2-anatase phase is excluded from the refinement again. In general terms, the a-axis appears to shrink for the discharged state compared to the charged states, whereas the b- and c-axes appear to increase. Only slight changes are observed for the monoclinic angle, β.

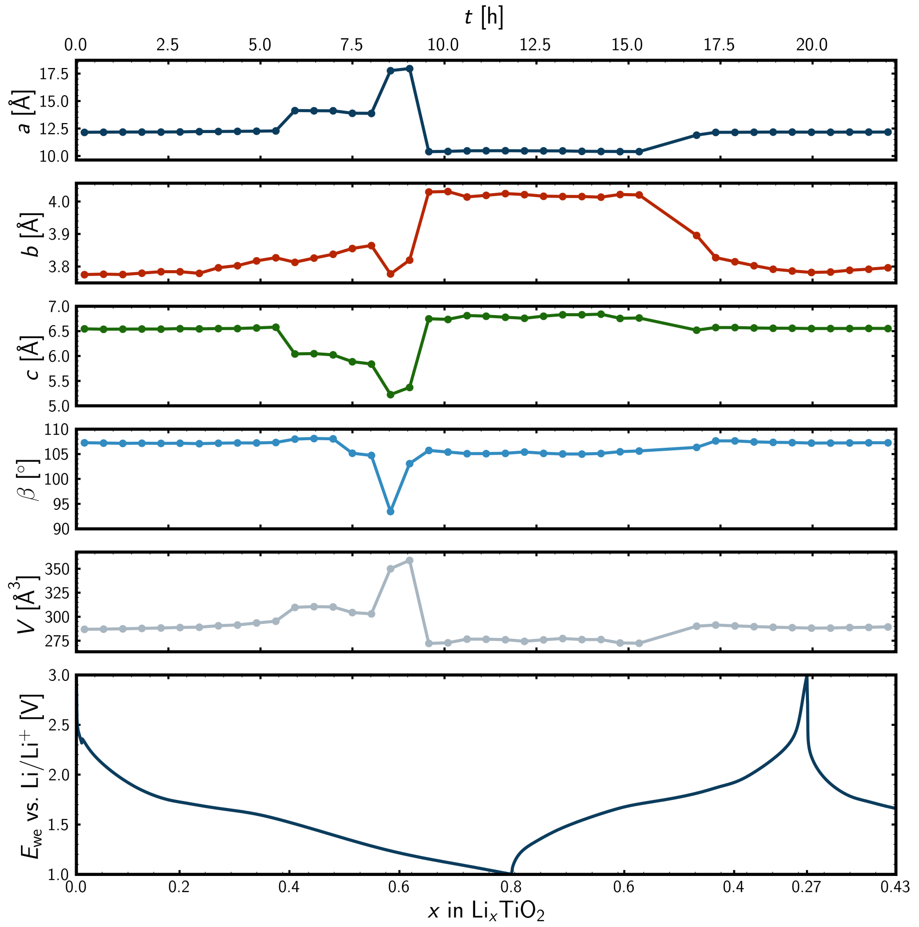

Fig. G5. Unit cell parameter values for the TiO_2-bronze phase from PDF modelling of the *operando* data, together with the Galvanostatic cycling.

TiO_2-anatase unit cell parameters

Fig. G6 displays the refined lattice parameters for the TiO_2-anatase phase. It is noted that the *a*-axis increases and the *c*-axis decreases monotonically during the initial discharge. During the last part of the *operando* experiment, the *a*-axis is at the level it ends at during the initial discharged, whereas the *c*-axis is more or less at the same lavel as for the initial discharge, though a decrease it observed for the former part of the charge process when the phase is included in the refinement.

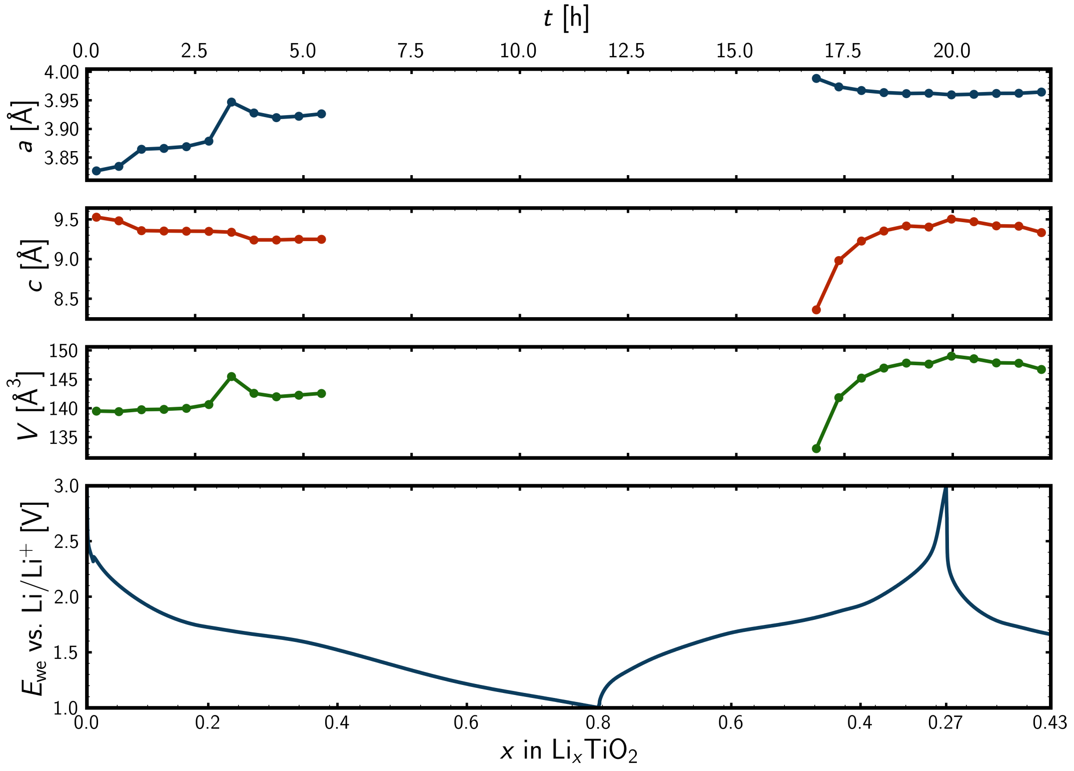

Fig. G6. Unit cell parameter values for the TiO_2-anatase phase from PDF modelling of the *operando* data, together with the Galvanostatic cycling.

Li_xTiO_2-bronze unit cell parameters

Fig. G7 displays the refined lattice parameters for the Li_xTiO_2-bronze phase. The refined values are observed to fluctuate a bit. However, the refined values appear to be stable around deep discharge and the first part of the charge process, where only the Li_xTiO_2-bronze and TiO_2-bronze phases are included in the refinement.

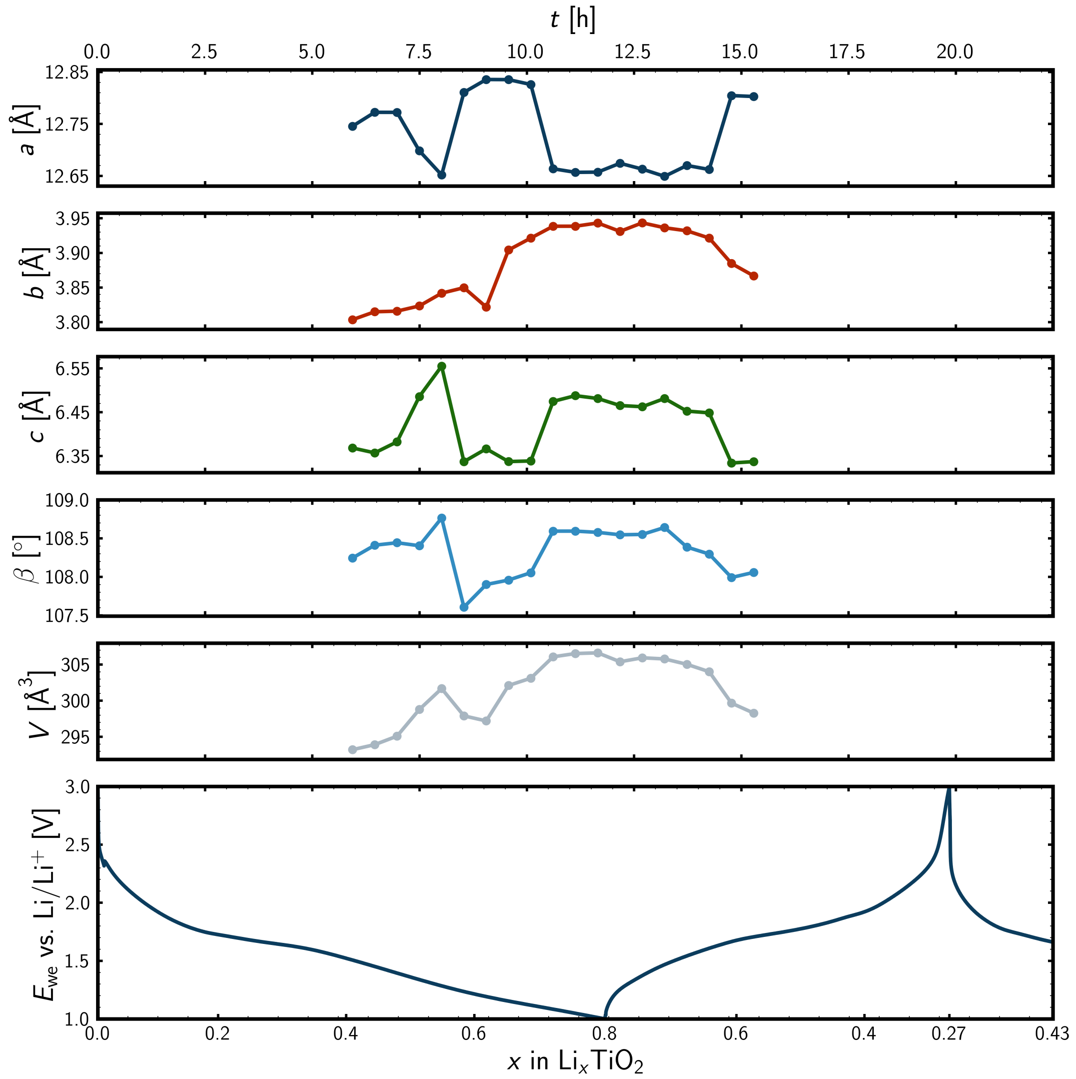

Fig. G7. Unit cell parameter values for the Li_xTiO_2-bronze phase from PDF modelling of the *operando* data, together with the Galvanostatic cycling.

Li_xTiO_2-anatase unit cell parameters

Fig. G8 displays the refined lattice parameters for the Li_xTiO_2-anatase phase. The refined values for the first four frames appear different from the latter. During the refinement of the first four frames, the TiO_2-anatase is also a part of the refinement. For the last two frames, the phase fraction is low, which results in the sudden jump for the refined values for the last two frames, where the Li_xTiO_2-bronze phase is included in the refinement.

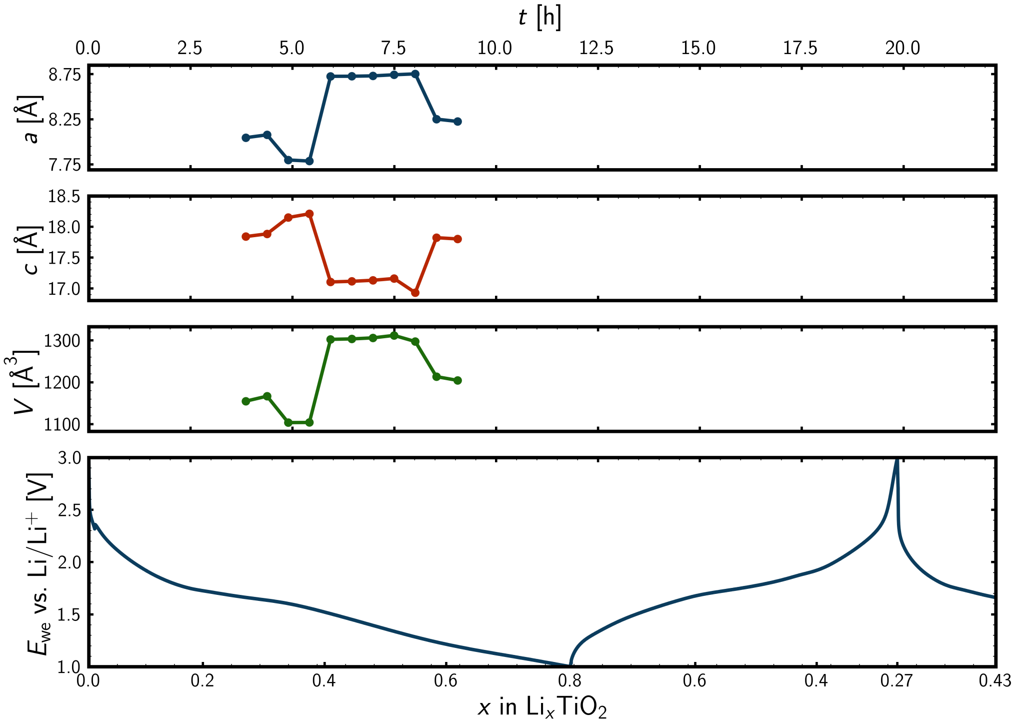

Fig. G8. Unit cell parameter values for the Li_xTiO_2-anatase phase from PDF modelling of the *operando* data, together with the Galvanostatic cycling.

Modified graphite unit cell parameter

Fig. G9 displays the refined lattice parameter for the modified graphite phase. As expected, the refined lattice parameter value is almost constant throughout the PDF modelling of the *operando* data.

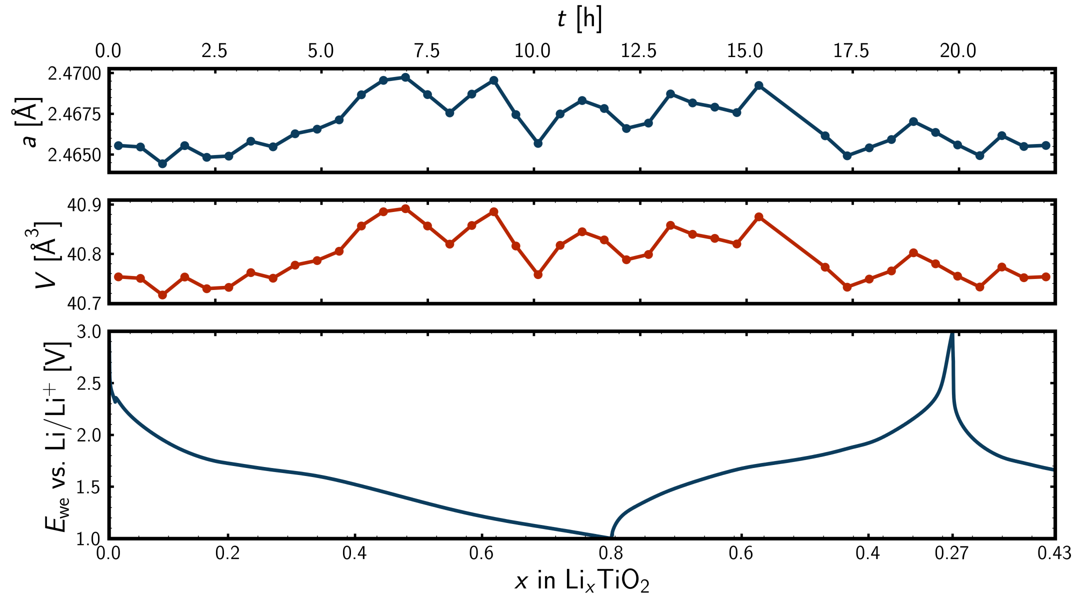

Fig. G9. Unit cell parameter values for the modified graphite phase from PDF modelling of the *operando* data, together with the Galvanostatic cycling.
